# Clinical care of childhood sexual abuse: a systematic review and critical appraisal of guidelines from European countries

**DOI:** 10.1016/j.lanepe.2024.100868

**Published:** 2024-02-21

**Authors:** Gabriel Otterman, Ulugbek B. Nurmatov, Ather Akhlaq, Laura Korhonen, Alison M. Kemp, Aideen Naughton, Martin Chalumeau, Andreas Jud, Mary Jo Vollmer Sandholm, Eva Mora-Theuer, Sarah Moultrie, Diogo Lamela, Nara Tagiyeva-Milne, Joanne Nelson, Jordan Greenbaum, the COST Action 19106 Research Team

**Affiliations:** a Barnafrid and Department of Biomedical and Clinical Sciences, Linköping University, Linköping, Sweden; b Division of Population Medicine, School of Medicine, Cardiff University, Cardiff, UK; c Institute of Business Management, Karachi, Pakistan; d National Safeguarding Service, Public Health Wales, Cardiff, UK; e Child Protection Unit - Department of General Pediatrics and Pediatric Infectious Diseases, Necker-Enfants malades Hospital, France; f Clinic for Child and Adolescent Psychiatry, Ulm University Clinics, Ulm, Germany; g Department of Forensic Sciences, Oslo University, Oslo, Norway; h Department of Pediatrics and Adolescent Medicine, Medical University of Vienna, Vienna, Austria; i Pediatric Trauma Services, Benioff Children's Hospitals- Oakland, Oakland, CA, USA; j Digital Human-Environment Interaction Lab (HEI-Lab), Lusófona University, Porto, Portugal; k Department of Education, Liverpool School of Tropical Medicine, Liverpool, UK; l Child and Adolescent Sexual Assault Treatment Service, Barnahus West, Saolta University Health Care Group, Galway, Ireland; m International Centre for Missing and Exploited Children, Alexandria, VA, USA

**Keywords:** Child maltreatment, Child sexual abuse, Clinical guidelines, Systematic review

## Abstract

**Background:**

The clinical management of Child sexual abuse (CSA) demands specialised skills from healthcare professionals due to its sensitivity, legal implications, and serious physical health and mental health effects. Standardised, comprehensive clinical practice guidelines (CPGs) may be pivotal. In this systematic review, we examined existing CSA national CPGs (NCPGs) from European countries to assess their quality and reporting.

**Methods:**

We systematically searched six international databases and multiple grey literature sources, reporting by the Preferred Reporting Items for Systematic Reviews and Meta-Analyses (PRISMA) standards. Eligible guidelines were CSA guidance from national health agencies or societies in 34 COST Action 19106 Network Countries (CANC), published between January 2012 and November 2022. Two independent researchers searched, screened, reviewed, and extracted data. NCPGs were compared for completeness with reference WHO 2017 and 2019 guidelines. We used the Appraisal of Guidelines for Research and Evaluation (AGREE II) to appraise quality and reporting. **PROSPERO**: CRD42022320747.

**Findings:**

Of 2919 records identified by database searches, none met inclusion criteria. Of 4714 records identified by other methods, 24 NCPGs from 17 (50%) of CANC countries were included. In 17 (50%) of eligible countries, no NCPGs were found. Content varied significantly within and between countries. NCPGs lacked many components in state-of-the art clinical practice compared to WHO reference standards, particularly in safety and risk assessment, interactions with caregivers, and mental health interventions. Appraisal by AGREE II revealed shortcomings in NCPG development, regarding scientific rigour, stakeholder involvement, implementation and evaluation.

**Interpretation:**

A notable number of European countries lack an NCPG; existing NCPGs often fall short. The healthcare response to CSA in Europe requires a coordinated approach to develop and implement high-quality CPGs. We advocate for a multidisciplinary team to develop a pan-European CSA guideline to ensure quality care for survivors.

**Funding:**

Funding was provided by the International Centre for Missing and Exploited Children.

**Research in context**

**Evidence before this study**

A 2021 systematic review of clinical practice guidelines (CPGs) for diagnosing child physical abuse in high-income countries revealed significant gaps in completeness and inconsistencies on key issues. This led us to investigate the evidence for national guidelines on the clinical management of child sexual abuse (CSA) in European countries. We conducted a preliminary focused search on Medline using 24 specific terms related to CPGs for clinical management of CSA without geographical or language restrictions (see Supplement 1) on 19 December 2021 which identified 490 records. Our search yielded no systematic reviews of guidelines from European countries on CSA clinical care. Studies indicate that healthcare professionals' readiness to address suspected CSA in Europe is frequently inadequate in terms of knowledge, skills, and confidence, with notable variations in clinical management practices. While the World Health Organization (WHO) had in 2017 and 2019 issued exhaustive CPGs for CSA care targeting a global audience, the publication and quality of corresponding national CPGs (NCPGs) by health agencies or academic societies in Europe was not known.

**Added value of this study**

Providing the most comprehensive analysis of national CPGs for CSA published in European countries, this systematic review involved a search of six international databases, as well as the grey literature. We aimed to identify NCPGs issued in 34 European COST Action 19106 Network Countries (CANC) over a ten-year period (2012–2022). The study introduced a critical assessment of guideline quality and clarity. We comprehensively compared 24 identified NCPGs against the WHO's 2017 and 2019 CSA guidelines, revealing both alignment and disparities between them. We used the Appraisal of Guidelines for Research & Evaluation (AGREE II) tool to evaluate the quality and methodological rigour of included NCPGs. No NCPGs were identified in 50% of CANC countries; 24 NCPGs were published in 17 (50%) countries. Considerable variation in the content of the identified NCPGs was revealed within and across countries. Existing NCPGs lacked many of the components included in state-of-the art clinical practice. Appraisal by the AGREE II tool revealed severe shortcomings in the development and implementation of NCPGs.

**Implications of all the available evidence**

Many countries in Europe lack a NCPG, and it is not known how widely implemented the NCPGs may be in countries in which NCPGs have been issued. In a context demanding cohesive, effective, and reliable healthcare responses to CSA, the significance of evidence-based, updated guidelines is paramount. The actionable recommendations ensuing from this research not only act as a roadmap for elevating the quality and consistency of CSA care but may also fill a substantial void from a healthcare management perspective. Our findings lead us to call for the convening of a group of multidisciplinary experts to develop a standardised pan-European guideline as a strategic method to ensure consistent, high-quality care for survivors in the region. The proposed guideline will need to consider factors like regional epidemiology, healthcare systems, and socioeconomic aspects unique to Europe. We recommend that the standardised guideline be accompanied by sustainably funded efforts to secure wide implementation, monitoring of its use and measurement of clinical outcomes.

## Introduction

Child sexual abuse (CSA) refers to the involvement of a child under the age of 18 in inappropriate sexual activities, which can occur with or without physical contact.^1^^,^^2^ In a 2011 global meta-analysis of CSA prevalence (using varied periods of prevalence), the combined rates in Europe were 13.5% (95% CI 11.0–16.5) for girls and 5.6% (95% CI 3.8–8.4) for boys.^3^ CSA is associated with a plethora of short and long-term adverse effects, including sexually transmitted infections (STI), traumatic injury, unwanted pregnancy, substance misuse, major depressive disorder, post-traumatic stress disorder (PTSD), complex PTSD, anxiety disorders, antisocial behaviours, commercial sex work, lower educational attainment, lower odds of being financially stable,^4^, ^5^, ^6^, ^7^, ^8^, ^9^ re-victimization, and difficulty forming and sustaining healthy relationships.^4^^,^^10^^,^^11^

When a healthcare professional identifies CSA or a child at risk, it is vital for the safety and wellbeing of the child that they respond appropriately. This includes obtaining a thorough history using a trauma-informed approach, documenting and interpreting historical and physical findings accurately, following best practices for medical management of STIs, offering physical health follow-up as well as referrals for culturally responsive, appropriate psychological support, assessment and treatment, and making necessary reports to authorities.^12^

To optimize the clinical management of CSA victims, healthcare professionals need assistance in the form of structured, comprehensive clinical practice guidelines (CPGs), defined as “statements that include recommendations, intended to optimize patient care, that are informed by a systematic review of evidence and an assessment of the benefits and harms of alternative care options.”^13^ CPGs ensure that patients receive uniform and high-quality care across settings and providers. They can improve patient outcomes, promote cost-effectiveness, provide a training tool for healthcare professionals, facilitate benchmarking for quality improvement, and support complex management by multidisciplinary teams. The World Health Organization (WHO) published CPGs on the initial response and intervention for CSA in 2017 and expanded their scope in 2019 by publishing guidelines on the identification, prevention of recurrence, initial response and intervention for all types of maltreatment.^14^^,^^15^ The latter document excluded material already covered by the 2017 guidelines. Both CPGs were based upon a rigorous review of literature and targeted the values and preferences of survivors, caregivers, and healthcare professionals, as well as practices concerning the reporting of suspected CSA to relevant statutory agencies.^16^

Several studies have reported that the preparedness and response of healthcare professionals to suspected CSA in Europe is often suboptimal regarding knowledge, skills, and confidence.^17^, ^18^, ^19^ Healthcare professionals may not be aware of the WHO guidelines, may choose not to adopt them, or may lack access to the guidelines in their language. They may also opt to use concurrent, less rigorous, or incomplete guidelines developed by scholarly organisations, regional and local authorities, or healthcare institutions.^20^

There are no previous systematic evaluations of the quality of content and reporting of national CSA guidelines in Europe. To address this knowledge gap, this systematic review has critically appraised existing national CPGs (NCPGs) issued within 34 countries represented in the EU COST Action 19106 network titled “Multi-Sectoral Responses to Child Abuse and Neglect in Europe: Incidence and Trends” (see Supplement 2).

## Methods

### General design

This systematic review was registered at the International Prospective Register of Systematic Reviews (PROSPERO CRD42022320747) and reported according to current international guidelines.^21^ A detailed SR protocol has been published previously elsewhere.^22^

### Search strategy and guidelines selection

Guidelines were identified in this review through a search of six international electronic databases, CINAHL, Embase, Medline, PsycINFO, TRIP, Web of Science, as well as searches of Google Scholar, GIN, WHO Global Health, SUMSearch2, the Health Management Information Consortium (HMIC), the National Institute for Health and Care Excellence (NICE, UK) (see Supplements 3–5). In addition, we searched the websites of government health agencies and academic societies within 34 COST Action 19106 Network Countries (CANC). Researchers and stakeholders from these nations have formed a network dedicated to improving multisectoral child abuse and neglect data collection across Europe. We also asked child abuse experts from each country to identify or confirm the lack of NCPGs.

The search terms applied were “child abuse” or “child sexual abuse” or “child sexual exploitation” or “sexual violence” or “sexual assault” or “rape” or “sex trafficking” or “sexual coercion”AND “diagnosis” or “diagnostic work up” or “investigation” or “management” or “assessment” or “clinical pathway” or “care path” or “critical pathway” AND “infant” or “child” or “young adult” or “adolescent” or “teenager” or “boy” or “girl” AND “clinical guideline” or “guideline” or “practice guideline” or “recommendation.” (see Supplements 3–5) Furthermore, we contacted subject matter experts to identify relevant guidelines or to confirm their absence in each of the 34 CANC countries: Albania, Austria, Belgium, Bosnia and Herzegovina, Bulgaria, Croatia, Cyprus, Denmark, Estonia, Finland, France, Germany, Greece, Hungary, Iceland, Ireland, Israel, Italy, Latvia, Lithuania, Malta, Moldova, the Netherlands, North Macedonia, Norway, Poland, Portugal, Romania, Slovenia, Spain, Sweden, Switzerland, Turkey and the United Kingdom.

### Selection process

Two authors searched databases and screen titles and abstracts independently for potentially eligible studies. Disagreement between researchers were resolved by consensus or arbitration involving a third author where necessary. Full texts of studies were retrieved for selected guidelines, and two authors evaluated whether these met inclusion/exclusion criteria. Disagreement was resolved by referral to a third author when necessary. A list of full-text records that did not meet criteria and the reasons for their exclusion are tabulated in Supplement 6.

### Inclusion and exclusion criteria for identified records

Guidelines were eligible for inclusion in the systematic review if they meet all the following criteria:

• Included specific guidance—that may include good practice statements—for healthcare professionals to recognise and appropriately respond to children (<18 years) who may have experienced CSA.
• Were published in peer-reviewed journals or in the grey literature (including government or health professional society websites).
• Represented the product of a national governmental agency or an academic society of health professionals located within the CANC.
• Were published between January 2012 and November 2022 (the time period of 10 years was determined based on the subject-matter experts' familiarity as clinicians with the publication and utilisation of CSA guidelines, and the estimated frequency of updates of guidelines required to maintain their validity in the face of developments in the underlying published evidence).^23^ Reports issued prior to this time may be outdated in certain topics such as diagnosis and treatment of infection. In addition, the beginning of the study period in January 2012 coincides with the introduction of significant legislation in the European Union in December 2011 of Directive 2011/93—Combatting the sexual abuse and sexual exploitation of children and child pornography, which specifies responsibilities of member states to ensure support of victims of CSA.^24^
• No language restrictions were applied.

Documents were excluded if they did not meet the above criteria, e.g., were research papers, systematic reviews, local or regional guidelines, institutional forms, or policy documents. Please see Supplement 6.

### Decision on the reference CSA guideline

We selected the WHO 2017 guidelines and the additional elements relevant to CSA in the WHO 2019 technical report due to their comprehensive and specific evidence-based recommendations, as well as guiding principles (GPs) and ‘good practice statements’ (GPS).^14^^,^^15^ Together, they encompass 101 recommendations, GPs, and GPS bullets. When deciding on the benchmark guideline, we appraised the reference WHO guidelines using the AGREE II tool (see below) and their high scores affirmed our decision to consider them the ‘gold standard’ in our analysis.^25^

### Data collection for content of NCPGs

To assess the content of each included guideline by comparison to the WHO 2017 and 2019 guidelines, we established the list of the items addressed in these two guidelines (see Supplement 7). The 2017 WHO guidelines contained 7 topic domains, which yielded 65 scored items; the included elements from the 2019 guidelines encompassed 4 topic domains and 36 scored items (101 total scored items).The final list of items encompassed the following 11 domains: child-or adolescent-centred care/first-line support; medical history, physical examination and documentation of findings; HIV post-exposure prophylaxis treatment and adherence; pregnancy prevention and clinical management; post-exposure prophylaxis for curable and vaccine-preventable STIs; psychological and mental health interventions in the short term and longer term; and ethical principles and human rights standards for reporting child or adolescent SA; initial identification; safety and risk assessment; interacting with caregivers; and guiding principles.

Non-English clinical guidelines were translated using readily available tools such as Google Translate, DeepL Translator and by consulting the research network. Two reviewers were paired and independently extracted data from included NCPGs and entered it into a customised extraction sheet (Supplement 7). Scoring of NCPG guideline content involved assigning each of the 101 items to one of three categories: “absent”, “partially present” and “present”. If a guideline mentioned material covered in a WHO recommendation or ‘good practice statements’ (GPS) bullet, but did not fully cover the content, it was scored as ‘partially present’. Total absence of, or complete coverage of recommendations or GPS elements were scored as ‘absent’ or ‘present’, respectively. The two reviewers provided justifications for each score, and copied verbatim text and page number where the information was located within the guideline. The reviewer pairs discussed and arrived at consensus on each of the 101 items; where consensus was lacking on a specific item, a third reviewer acted as arbitrator.

We converted categorical data to numeric data, assigning “absent” as 0.0 value, “partially present” as 0.5 and “present” as 1 value (see Table 1). We calculated total scores for each of the 11 domains covered in the WHO guidelines and a total composite score of the 101 items. The domain scores were tabulated, and colour coded according to their place above or below the mean domain score.

**Table 1:** Descriptive characteristics of included CSA NCPGs (n = 24) when compared to WHO domains.




The guidelines are presented in descending order based on the total composite scores.

The cumulative scores for different topic domains are presented along with illustration of domain quality with colour coding. Dark green = max score; light green = mean or score in between mean and max; orange = score between mean and min; red = min.

Reviewers extracted any identified definitions of CSA as stated in the WHO reference guidelines and included NCPGs (Supplement 8).

### Quality and reporting appraisal of WHO and national guidelines

We used the Appraisal of Guidelines for Research & Evaluation (AGREE II) tool to evaluate the quality, methodological rigour and transparency of included national clinical guidelines (NCPGs).^25^ The AGREE II instrument consists of a total of 23 items in six domains: ‘Scope and purpose’, ‘Stakeholder involvement’, ‘Rigour of development’, ‘Clarity of presentation’, ‘Applicability’, and ‘Editorial independence’. Each item was rated on a seven-point scale from 1 (strongly disagree) to 7 (strongly agree).

### Quality change of guidelines over time

To assess the impact of the first WHO CSA guidelines published in 2017 on the quality of NCPGs, we compared AGREE II scores of NCPGs published between 2014 and 2017 to those issued from 2018 up to 2022 using the Student's t-test for mean quality scores, analysed with SPSS v21.

### Data synthesis

We tallied the number of CANC countries in which at least one CSA guideline was available or lacking and described the general characteristics of the guidelines. We reported the frequency of full or partial alignment of items within each guideline relative to the WHO reference standard guidelines. We reported the results of the quality appraisal for the included guidelines.

This review adhered to the registered and published protocol,^22^ except for using the GRADE (Grading of Recommendations, Assessment, Development, and Evaluations) framework. We opted to omit GRADE because, although it's effective when interpreting results from systematic reviews of interventions, the AGREE II tool is validated for reviewing other topics included in the NCPGs.

### Role of the funding source

The funder of the study had no role in study design, data collection, data analysis, data interpretation, or writing of the report.

## Results

**Fig. 1:**
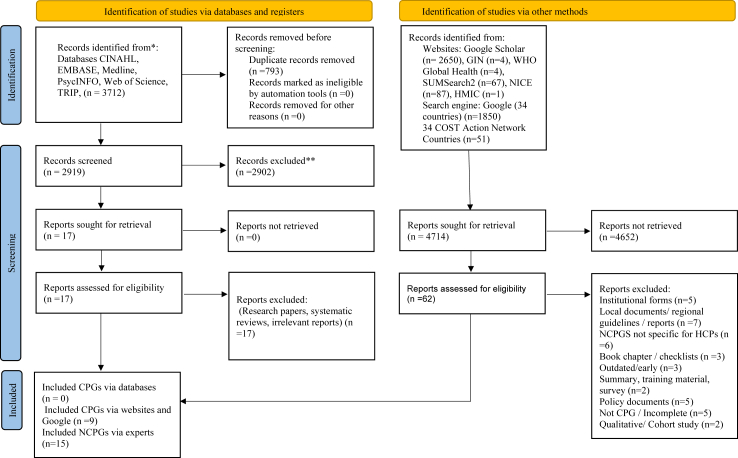
**Study selection**.

Our searches identified 8426 potentially relevant documents; following de-duplication, 7633 publications were included for screening (Fig. 1). None of the 2919 records identified via international database searches fulfilled the inclusion criteria. Of the additional 4714 reports identified via other methods, 24 CSA NCPGs satisfied our inclusion criteria and were included in our systematic review (Table 1).^26^, ^27^, ^28^, ^29^, ^30^, ^31^, ^32^, ^33^, ^34^, ^35^, ^36^, ^37^, ^38^, ^39^, ^40^, ^41^, ^42^, ^43^, ^44^, ^45^, ^46^, ^47^, ^48^, ^49^, ^50^ Nine NCPGs were identified using websites and Google searches, and 15 were identified through CANC experts.

**Fig. 2:**
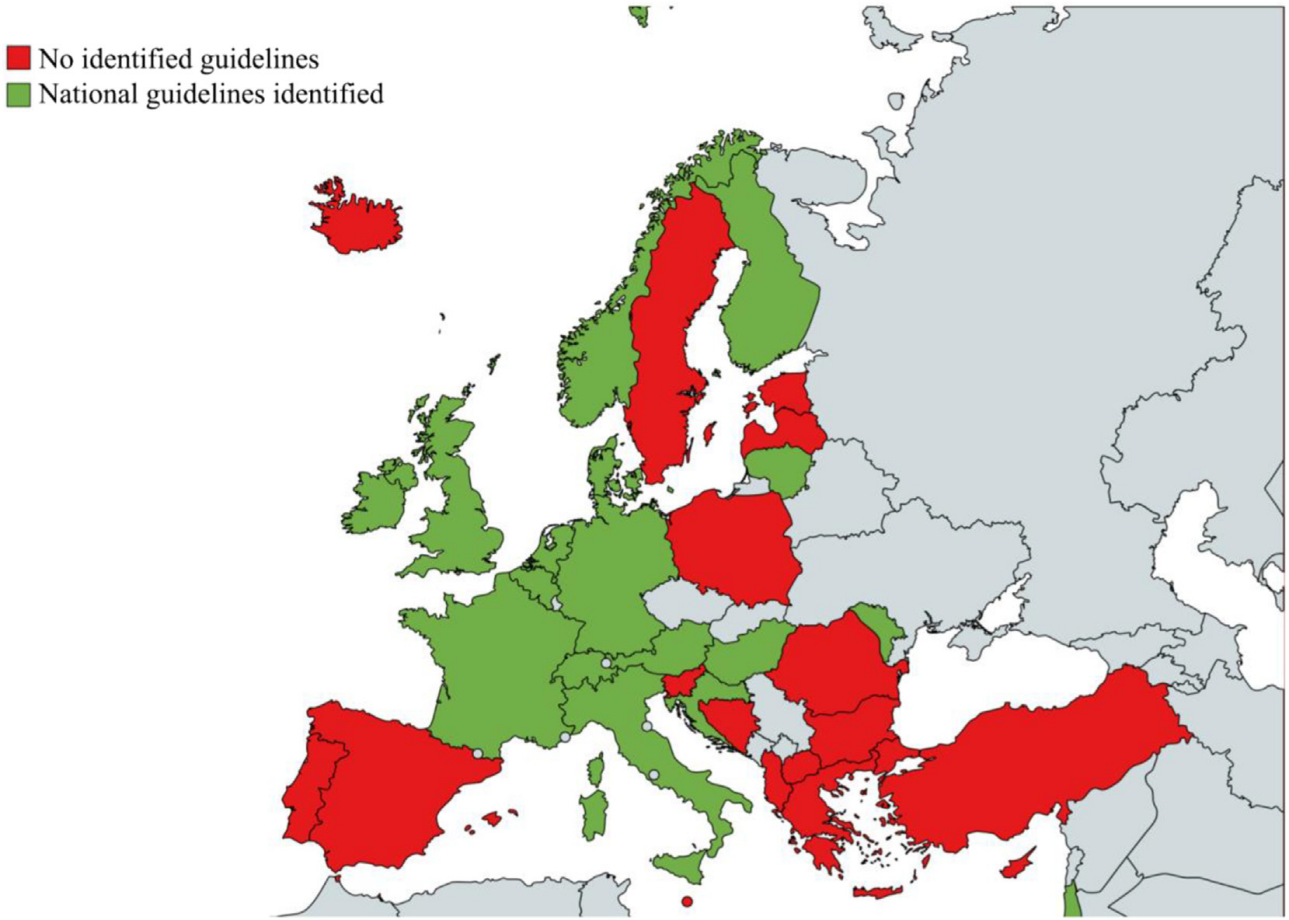
COST Action 19106 eligible countries (n = 34) with (green; n = 17) and without (red; n = 17) identified national clinical practice child sexual abuse guidelines.

In each of five countries, Belgium, Italy, Lithuania, the Netherlands, and the UK, two to three NCPGs were included. In total, NCPGs were identified in 17 of the 34 CANCs corresponding to 50% of the eligible CANC countries (Fig. 2).

Of the included NCPGs, four were published in English; twenty NCPGs were translated from the language of publication to English. Eleven NCPGs addressed child maltreatment, encompassing physical, emotional, and sexual abuse. Meanwhile, thirteen NCPGs focused on CSA, including one that specifically targeted online CSA (Table 1). Of the 24 NCPGs, 15 focused on minors while nine covered both adults and children.

### Appraisal of the guideline contents

The completeness and clarity of the identified guidelines were compared against the WHO ‘gold standard’. Comparison of NCPGs to content domains showed considerable variation between countries and even between NCPGs within a single country (Table 1). No specific domain was consistently well addressed by all NCPGs, except that focusing on child-centred care (14/24 had domain scores above the mean).

**Fig. 3:**
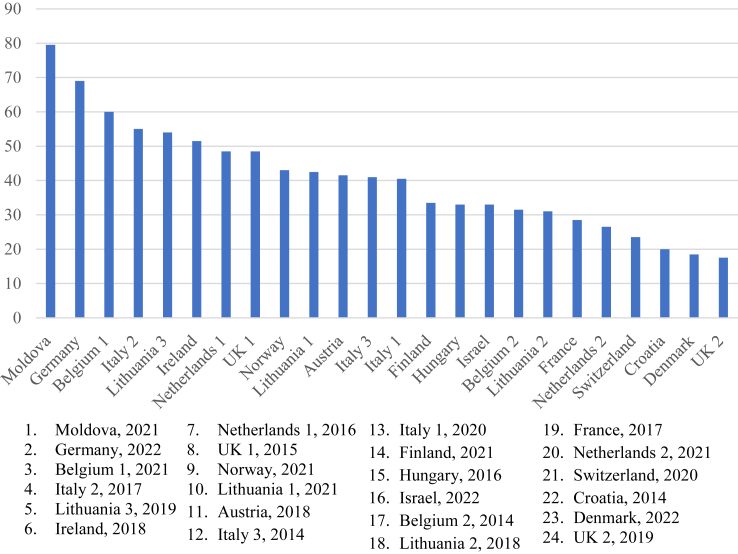
Total points scored for 101 WHO items.

Comparison against WHO standards revealed significant gaps in critical components of care. Overall, NCPGs from the following three countries had the highest total composite score (max 101 points): Moldova = 79.5; Germany = 69.0; and Belgium 1 = 60.0 (Table 1). The three NCPGs with the lowest composite scores were Croatia = 20.0; Denmark = 18.5; and UK, 2019 = 17.5 (Fig. 3). Only 6 of the 24 (25%) guidelines had a composite score above 50.

The 3 domains with the lowest scores among NCPGs involved mental health intervention (22/24 NCPGs scored below the mean for the domain), safety/risk assessment (21/24 scored below mean) and interactions with caregivers (20/24 below mean). Altogether, 6/24 NCPGs had scores of ‘0’ for 4 of the domains; 17/24 had at least 6 domains with overall scores less than the mean.

The differences extended across the 11 domains when compared to the WHO guidelines. Particularly, domains related to HIV prophylaxis and treatment (lacking in 37.5% of the guidelines), pregnancy prevention and clinical management (lacking in 37.5%), post-exposure STI prophylaxis (lacking in 33.3%), and psychological and mental health interventions (lacking in 42.7%).

Table 2 lists the individual WHO bullets most commonly absent from NCPGs (at least 80% absent rate) and bullets most commonly present in the NCPGs (0–20% absent rate). Commonly absent items tended to involve mental health interventions, assessment measures, and provider bias. In contrast, items commonly addressed in the NCPGs included principles of trauma-informed care and comprehensive, well-documented evaluations.

**Table 2:** WHO bullets most/least commonly absent from NCPGs.

Item	Absent %	WHO bullet	Domain
Bullets most commonly ABSENT from NCPGs with at least 80% absent rate			
48	96%	Psychological debriefing should not be used in an attempt to reduce the risk of post-traumatic stress, anxiety or depressive symptoms.	F. Psychological and mental health interventions in the short term and longer term, WHO 2017
91	96%	Take care not to allow relationships with other family members to interfere with the ability to consider maltreatment.	D. Interacting with Caregivers, WHO 2019
28	92%	Not conducting virginity testing (two-finger test or per-vaginal examination), as it increases distress and does not indicate whether or not abuse took place.	B. Medical history, physical examination and documentation of findings, WHO 2017
70	92%	Written information on child maltreatment should be available in health-care settings in the form of posters, and pamphlets or leaflets *(with appropriate warnings about taking them home in case that could compromise safety).*	A. Initial identification, WHO 2019
83	92%	Treat instruments as a tool to enhance or expand clinical judgement, not as a substitute for clinical judgement.	C. Safety and Risk, WHO 2019
49	88%	CBT with a trauma focus should be considered for children and adolescents who have been sexually abused and are experiencing symptoms of PTSD.	F. Psychological and mental health interventions in the short term and longer term, WHO 2017
82	88%	Be aware of the many factors that influence the risk of recurrence that may not be accounted for by assessment instruments.	C. Safety and Risk, WHO 2019
23	83%	Offering choice in the sex of the examiner, where possible.	B. Medical history, physical examination and documentation of findings, WHO 2017
50	83%	When safe and appropriate to involve at least one nonoffending caregiver, CBT with a trauma focus should be considered for both: (i) children and adolescents who have been sexually abused and are experiencing symptoms of PTSD; and (ii) their non-offending caregiver(s).	F. Psychological and mental health interventions in the short term and longer term, WHO 2017
61	83%	Address health-care providers' beliefs and values that can adversely affect their reporting practices.	G. Ethical principles and human rights standards for reporting child or adolescent sexual abuse, WHO 2017
Bullets most commonly PRESENT in the CGs (0–20% absent rate)			
4	17%	Taking actions to enhance their safety and minimize harms, including those of disclosure and, where possible, the likelihood of the abuse continuing, this includes ensuring visual and auditory privacy.	A. Child- or adolescent-centred care/first-line support, WHO 2017
6	17%	Providing age-appropriate information about what will be done to provide them with care, including whether their disclosure of abuse will need to be reported to relevant designated authorities.	A. Child- or adolescent-centred care/first-line support, WHO 2017
15	17%	Conducting a comprehensive assessment of their physical and emotional health, in order to facilitate appropriate decisions for conducting examinations and investigations, assessing injuries and providing treatment and/or referrals.	B. Medical history, physical examination and documentation of findings, WHO 2017
30	17%	Using a structured format for recording the findings.	B. Medical history, physical examination and documentation of findings, WHO 2017
33	17%	Recording a detailed and accurate description of the symptoms and injuries.	B. Medical history, physical examination and documentation of findings, WHO 2017
53	17%	Assessing the implications of reporting for their health and safety and taking steps to promote their safety.	G. Ethical principles and human rights standards for reporting child or adolescent sexual abuse, WHO 2017
59	17%	Be aware of any legal requirements to report known or suspected cases of abuse.	G. Ethical principles and human rights standards for reporting child or adolescent sexual abuse, WHO 2017
75	17%	Assessing the child or adolescent's physical and emotional safety needs.	C. Safety and Risk, WHO 2019
97	17%	Provide information that is appropriate to age and developmental stage as well as to other considerations (e.g., sex, race, ethnicity, religion, sexual orientation, gender identity, disability and socioeconomic status). This requires tailoring the information that is offered and how it is delivered (e. g. in choice of words or language, use of visual aids) to the child's or adolescent's age and developmental stage, including their cognitive, behavioural and emotional maturity to understand the information.	E. Guiding principles, WHO 2019
99	17%	Respect the autonomy and wishes of children or adolescents (e.g, not forcing them to give information or be examined) while balancing this with the need to protect their best interests (e.g., protect and promote their safety). In situations where a child's or adolescent’s wishes cannot be prioritized, the reasons should be explained to the child or adolescent before further steps are taken.	E. Guiding principles, WHO 2019
1	13%	Listening respectfully and empathetically to the information that is provided.	A. Child- or adolescent-centred care/first-line support, WHO 2017
7	8%	Attending to them in a timely way and in accordance with their needs and wishes.	A. Child- or adolescent-centred care/first-line support, WHO 2017
95	8%	Provide sensitive care: children and adolescents who disclose maltreatment including abuse need to be listened to attentively, without interpreting or judging their account, even when it might differ from that of the accompanying caregivers. Children and adolescents should be offered an empathetic and non-judgemental response that reassures them that they are not to blame for the maltreatment including abuse and that they have acted appropriately in disclosing it.	E. Guiding principles, WHO 2019
79	4%	Involving other relevant agencies, in consultation with the child or adolescent, if the child's safety is at risk. Information including contact details of relevant agencies should be made available to health care providers. In some settings no legal mechanism may be available to separate children from perpetrators of maltreatment in their current living arrangements or removing the child or adolescent may expose them to an even less safe environment. In such situations careful and frequent follow-up by health workers will be particularly important.	C. Safety and Risk, WHO 2019
63	0%	Work with other agencies or institutions, including child protection and police services, in order to coordinate an appropriate response.	G. Ethical principles and human rights standards for reporting child or adolescent sexual abuse, WHO 2017

### Quality assessment

Table 3 lists the standardized scores pertaining to each domain of AGREE II. The standardised scores for each domain were highly dispersed (Supplement 9–Fig. 4). There were high median values for the domains ‘scope and purpose’ (Mdn. = 69.0; IQR = 57.3–93.5) and ‘clarity of presentation’ (Mdn. = 70.5; IQR = 53.7–82.5), followed by ‘stakeholder involvement’ (Mdn = 47.0; IQR = 28.0–75.2). ‘Rigour of development’ (Mdn. = 22.0; IQR = 4.2–46.0) and ‘applicability’ (Mdn. = 24.0; IQR = 14.0–38.0) had low median values. However, the Moldova guideline was the outlier in the ‘applicability’ domain. Half of the guidelines did not follow the attributes of ‘Editorial Independence’ (Mdn. = 0.0; IQR = 0.0–48.0).

**Table 3:** Standardized scores by domains of AGREE II.

S.No.	Guidelines	Country	Scope and Purpose (%)	Stakeholder Involvement (%)	Rigour of Development (%)	Clarity of Presentation (%)	Applicability (%)	Editorial Independence (%)
1.	(A. Ciresa-König et al., 2021)	Austria	78	42	9	72	13	0
2.	(Keygnaert I. et al., 2021)	Belgium	33	17	17	72	38	0
3.	(N. Dekker, K. SmetS, & PeremaNS, 2014)	Belgium	92	78	43	56	21	25
4.	(Croatian_Government, 2014)	Croatia	56	44	4	22	25	0
5.	(Lassen, Christiansen, Jørgensen, & Berg, 2022)	Denmark	47	28	1	53	6	0
6.	(Luoma, Joki-Erkkilä, & Taskinen, 2021)	Finland	36	31	13	56	19	0
7.	(Dhénain, 2014; HAS, 2017)	France	94	50	28	83	23	50
8.	(Blesken et al., 2022)	Germany	100	92	79	92	27	100
9.	(Keller et al., 2016)	Hungary	69	64	49	47	52	42
10.	(Team, 2018)	Ireland	89	42	38	81	52	0
11.	(health.gov.il, 2022)	Israel	61	3	1	50	17	0
12.	(Angeletti et al., 2020)	Italy	69	31	32	75	38	0
13.	(Longo, Cremonesi, Pitidis, Santi, & Sparaco, 2017)	Italy	81	67	34	53	19	0
14.	(Orsini, Pagano, Raciti, & Seniga, 2014)	Italy	67	67	0	58	19	0
15.	(Dulkys, 2021)	Lithuania	67	28	3	72	25	0
16.	(Adomaitienÿ et al., 2019)	Lithuania	67	61	24	81	46	25
17.	(Adlienÿ et al., 2018)	Lithuania	36	25	5	64	4	21
18.	(Moldova, 2021)	Moldova	94	83	47	94	90	50
19.	(FMG, 2021)	Netherlands	64	28	20	56	13	0
20.	(Teeuw, Langendam, & Boseloos, 2016)	Netherlands	100	100	80	86	33	67
21.	(helsebiblioteket, 2021)	Norway	42	28	7	44	10	0
22.	(Lips, Wopmann, Jud, & Falta, 2020)	Switzerland	86	50	1	69	25	50
23.	(NICE, 2019)	United Kingdom	100	100	98	100	60	100
24.	(RCPCH, 2015)	United Kingdom	100	78	85	100	8	25
	**Mean Score**		72	51.5	29.9	68.1	28.4	23.1
	**Reference Guidelines (WHO 2017 and 2019)**		**97**	**92**	**92**	**94**	**92**	**88**

### Quality change of guidelines over time

Comparing the AGREE II scores of NCPGs published before and after the issuance of WHO guidelines in 2017, we found statistically significant changes only in the ‘stakeholder involvement’ domain (p = 0.027). However, the mean score in this domain was higher in 2014–2017 group than in the 2018–2022 group (Supplement 10–Table 4).

## Discussion

There were four major findings in this systematic review of NCPGs in 34 countries within the European Region: 1) In 50% of eligible countries, no NCPGs were identified; 2) There is considerable variation in the content of NCPGs within and across countries; 3) Existing guidelines lack many of the components included in state-of-the art clinical practice; 4) Quality appraisal revealed severe shortcomings in the development and strategic implementation of NCPGs.

Healthcare professionals in countries without NCPGs may rely on guidance from the WHO or other country/regional standards or may use local or institutional protocols. Alternatively, they may lack any formal guidance. They may decide a specific NCPG is unnecessary since any patient with suspicions of CSA is, by law or policy, directed to a government forensic institute. In the latter situation, however, an NCPG would still be useful for hospitals and clinics so that staff would be aware of risk factors and possible indicators of CSA, familiar with the process of reporting to authorities and making applicable referrals for specialist forensic or multidisciplinary evaluation, and aware of community resources that provide health and mental health follow-up services.

The NCPGs showed considerable variation in their content when compared to one another. When compared to the WHO guidelines, the differences extended across the 11 domains but were particularly marked for pregnancy and infection management domains. There are several possible explanations for these findings. The scope, purpose, target audience, and settings of the NCPGs varied. Some authors aimed to create general NCPGs limited to summary guidance, while others provided detailed recommendations. Five countries had two or three NCPGs identified with divergent aims and objectives. Some guidelines directed healthcare professionals to specific national or international guidelines for HIV and STI testing/treatment rather than including it in the NCPG. Others lacked a discussion of specific after-care options, including mental health care. Extensive research has documented the short- and long-term adverse effects of CSA on mental health^51^, ^52^, ^53^ and the effectiveness of psychological support and therapy.^54^^,^^55^ Healthcare professionals need to attend to these needs through psychoeducation on child traumatic stress,^56^ and referral to mental health aftercare services. The WHO guidelines are quite specific in discussing mental health therapy, emphasizing cognitive behavioural therapy with a trauma focus. These forms of therapy may not be validated in all CANCs and may not be widely accepted, leading to low scores in the WHO domain. Further, healthcare professionals in CANC countries may refer patients for mental health services that involve alternative methods of support. Additional research is needed to answer this question.

Three of the ten least commonly included bullets involved healthcare professionals' beliefs and potential biases. Most NCPGs did not admonish healthcare professionals to ‘take care not to allow relationships with other family members to interfere with the ability to consider maltreatment’ or ‘address health-care providers’ beliefs and values that can adversely affect their reporting practices.’ Pre-conceived notions about gender roles, including the victimization of boys^57^^,^^58^; and ethnic, racial, and socioeconomic biases may preclude recognition of at-risk children or lead to victim-blaming rather than victim-serving responses.^59^^,^^60^ Children's rights,^61^ especially the right to care free of bias and discrimination, are a critical component to be addressed by future NCPGs. Stigma experienced by patients and families has been shown to cause significant distress during health visits. It may prevent some children and families from seeking healthcare for sexual violence, thus jeopardizing their health and wellbeing.^62^^,^^63^

Analysis of the WHO bullets most cited in NCPGs revealed a strong representation of trauma-informed,^12^ rights-based principles^61^ for patient interaction. This finding is important in that, while quantitative validation of the trauma-informed approach is limited,^64^ qualitative studies of survivors of sexual violence consistently demonstrate the benefits of this strategy in facilitating patient trust in the clinician and encouraging open discussion of patient needs.^62^^,^^63^^,^^65^^,^^66^ Not surprisingly, other frequently mentioned items refer to practices that are part of routine clinical care, such as conducting a comprehensive assessment of physical and emotional health to guide examinations, treatment, and referrals, as well as adherence to mandatory reporting laws. These results are similar to findings from a recent systematic review of studies and protocols addressing treatment strategies and medical interventions related to CSA.^67^ In contrast, guidelines published by a European forensic medicine network in 2018 focused on the gynaecological medical exam of adults with limited discussion of a distinct trauma-informed approach for the examination of children.^68^

There were severe shortcomings in the development and implementation of NCPGs. The NCPGs scored relatively low in the quality assessment domain of ‘scientific rigour’. A solid evidence-base for NCPGs is critical and requires scientific rigour in identifying and assessing current research on CSA.^68^^,^^69^ Evidence synthesis through systematic reviews and meta-analyses provide the basis for clinical recommendations. The WHO guidelines employed comprehensive systematic literature reviews, largely absent from the NCPGs identified in our study.

The reasons for the lack of scientific rigour in NCPG development are unclear. The process is time and labour-intensive and may require financial and human resources unavailable to NCPG work groups. Governmental priorities may preclude adequate funding. Alternatively, it is possible that some or all the activities were completed but were not documented in the NCPGs. Many NCPGs had very limited sections describing their methodology. Future NCPGs would benefit from a clear, comprehensive explanation of methodology and a rigorous scientific approach.^14^^,^^15^

Quality appraisal of the NCPGs revealed also low scores on ‘stakeholder involvement.’ Evidence-based medicine incorporates patient values and needs, best determined by survivor involvement in guideline production.^69^ In the field of child protection, increasing emphasis is being placed on the importance of obtaining input from children^61^ and individuals with lived experience of sexual violence, as well as representatives of a range of professions involved with the recognition and response to CSA.^70^ Unfortunately, this is not reflected in our comparison of NCPG scores on stakeholder involvement across two time periods.

The low mean score on the ‘applicability’ domain is notable but perhaps not surprising. The criteria for this domain address activities related to NCPG implementation and evaluation. It may well be that working groups developing their NCPG did not consider these factors as within the scope of the document. It is critical for NCPG implementation to be organized, widespread, and accompanied by systematic monitoring for fidelity and use. Clinical outcomes should be evaluated accounting for type of CSA exposure, clinical and mental health sequelae, or signs and symptoms. The implementation rates and clinical outcomes of existing NCPGs are unknown and should be the subject of future research.

There is no universally accepted definition of CSA, but that published by the WHO is widely recognised. The definitions cited in NCPGs appeared to vary in accordance with the distinct scopes of the guidelines and relevant laws, where some guidelines had a more legal or forensic focus (Supplement 8). This variation was expected and did not change our analysis. However, a consensus on the definition and comprehensive scope of a guideline on the clinical care of children who experience CSA is desirable for optimal medical management, research and epidemiology.

### Strengths and limitations of this work

The strengths of this systematic review are the comprehensiveness of the searches and rigorous assessment of the quality and reporting. However, there are several limitations of this review. Despite our very comprehensive, multifaceted search strategy, it is possible that we did not identify some NCPGs. In some countries, healthcare is organized at the state, provincial, or municipal level, so our search strategy may miss guidelines used in practice. In addition, some countries may simply rely on clinicians using the WHO guidelines and may have no formally recognized CPG. A further limitation involves the subjective nature of evaluating whether a given bullet point in the WHO guidelines was ‘present’, ‘partially present’ or ‘absent’. To address this, we decided to limit the scoring options to 3, acknowledging that a ‘partially present’ score might encompass text that only minimally addressed the point to be considered or nearly all the relevant content. We also ensured that differences in scoring between paired reviewers were reviewed and a consensus score was reached. We did not need to resort to a 3rd party review as consensus was possible for all points. Some NCPGs were written in languages other than English, so were translated using readily available translation applications. Nuances of included standards may have been lost in translation. We minimized the impact of potential translation errors by assigning reviewers fluent in the publication language.

### Conclusions and recommendations for future work

Our systematic review and appraisal of NCPGs across 34 countries in the European Region revealed that half of the countries lack guidelines, there is significant inconsistency in their content, they often miss key components of advanced clinical practice, and there are major deficiencies in their development and strategic implementation. These deficiencies may lead to inconsistent and potentially suboptimal care of European children who experience CSA. While systematic and monitored efforts to expand use of the WHO guidelines throughout Europe may help ensure consistent and effective care, we recommend convening a group of multidisciplinary European experts and individuals with lived experience to synthesise the available evidence and systematically arrive at a consensus on a definition of CSA, recommendations and best practice. This would include a review of existing publications such as the WHO reference guidelines to ultimately create a set of evidence-based guidelines for CSA with implementation support and a monitoring plan specific to the European Region. The guidelines would accommodate the resource conditions, laws, policies, and stakeholders of Europe, and be subject to periodic updates. To our knowledge, there is no scheduled update for the WHO guidelines. The pan-European guidance should encompass a comprehensive, holistic, trauma-informed, rights-based physical health and mental healthcare response to all types of CSA. Rigorous scientific methodology should be used to design the standardized guideline. The centrally developed guideline should be adapted to address specific cultural, social, economic, and legislative conditions in each country.

## Contributors

GO and JG designed the study. UN, GO, and JG devised the methodology. UN and AA built and executed the searches. JG and GO evaluated full-text reports for inclusion. All authors (JG, GO, UN, AA, LK, AMK, AN, MC, AJ, MJVS, EMT, SM, DL, NTM, JKN) participated in data extraction and guideline appraisals. AA and UN conducted the statistical analysis. JG and GO drafted the manuscript with substantive edits for scientific content by UN, AA, LK, AMK, AN, MC, AJ and MJVS. All authors reviewed and approved the final version.

## Data sharing statement

Post-publication, the study data will be made available to researchers upon request (email: gabriel.otterman@liu.se).

## Editor note

The Lancet Group takes a neutral position with respect to territorial claims in published maps and institutional affiliations.

## Declaration of interests

None declared.

## Appendix A Supplementary data

Translations abstract
