# Supplementary material for: Clinical care of childhood sexual abuse: a systematic review and critical appraisal of guidelines from European countries
Source: Lancet Reg Health Eur. 2024 Feb 21;39:100868. doi: 10.1016/j.lanepe.2024.100868 (PMC10899013; doi:10.1016/j.lanepe.2024.100868)
Supplement: Translations abstract [file mmc1.docx]

This translation in Finnish was submitted by the authors and we reproduce it as supplied. It has not been peer reviewed. Our editorial processes have only been applied to the original abstract in English, which should serve as reference for this manuscript.

**Lapsuuden seksuaalisen hyväksikäytön kliininen tutkiminen ja hoito: systemaattinen katsaus Eurooppalaisten maiden hoitosuosituksiin ja niiden kriittinen arviointi**

**ABSTRACT:**

**Tausta:** Lapsiin kohdistuvan seksuaalisen hyväksikäytön tutkiminen ja hoito vaatiin terveydenhuollon henkilökunnalta erikoisosaamista aiheen arkaluontoisuuden, siihen liittyvien oikeudellisten kysymysten ja vakavien fyysisten ja psyykkisten seurauksien vuoksi. Yhtenäisillä ja kattavilla hoitosuosituksilla voi olla tärkeä merkitys. Tässä systemaattisessa katsauksessa tutkittiin Eurooppalaisten maiden olemassaolevia kansallisia kliinisiä hoitosuosituksia ja arvioitiin niiden laatua ja sisältöjä.

**Menetelmät:** Teimme systemaattisen haun käyttäen kuutta kansainvälistä tietokantaa ja useita harmaan kirjallisuuden tietokantoja PRISMA (Preferred Reporting Items for Systematic Reviews and Meta-Analyses)-standardeja noudatten. Katsaukseen hyväksyt julkaisut olivat kansallisia ohjeita, jotka olivat kansallisten terveysvirastojen tai -yhteisöjen laatimia ja julkaistu jossakin 34:sta COST- 19106 maasta (CANC) tammikuun 2012 ja marraskuun 2022 välisenä aikana. Kaksi toisistaan riippumatonta tutkijaa tekivät haut sekä seuloivat ja arvioivat julkaisut ja poimivat niistä tiedot. Kansallisia hoitosuosituksia verrattiin WHO:n vuonna 2017 ja 2019 julkaisemiin ohjeisiin. Laadun ja raportoinnin arvioinnissa käytettiin Appraisal of Guidelines for Research and Evaluation (AGREE II) -arviointimenetelmää.

**Tulokset:** Yksikään tietokantahakujen perusteella tunnistetuista 2919:sta julkaisusta ei täyttänyt inkluusiokriteerejä. Muilla menetelmillä löydetyistä 4 714:sta julkaisusta otettiin mukaan 24 kansallista ohjetta 17:stä (50 %) CANC-maasta. Kelpoisuusehdot täyttävistä maista 17:stä (50 %) ei löytynyt yhtään kansallista hoitosuositusta. Sisältö vaihteli merkittävästi maiden sisällä ja niiden välillä. WHO:n ohjeisiin verrattuna kansallisista hoitosuosituksista puuttui monia käypä hoidon osia koskien erityisesti turvallisuutta ja riskien arviointia, vuorovaikutusta vanhempien kanssa ja mielenterveysinterventioita. AGREE II -arviointi paljasti puutteita kansallisten hoitosuositusten kehittämisessä tieteellisen tarkkuuden, sidosryhmien osallistamisen sekä täytäntöönpanon ja arvioinnin osalta.

**Päätelmät:** Huomattava määrä Euroopan maita ei ole laatinut kansallisia hoitosuunnitelmia, ja olemassa ovat usein puutteellisia. Terveydenhuollon kyky tutkia ja hoitaa lapsia, jotka ovat kokeneet seksuaalista hyväksikäyttöä edellyttää Euroopassa koordinoitua lähestymistapaa korkealaatuisten hoitosuositusten laatimiseksi ja täytäntöönpanemiseksi. Suosittelemme monialaisen ryhmän perustamista, joka voisi laatia yleiseurooppalaisen hoitosuosituksen. Näin voitaisiin varmistaa seksuaalista hyväksikäyttöä kokeneiden lasten laadukas kliininen tutkiminen ja hoito.

**Rahoitus:** Tutkimuksen rahoitti International Centre for Missing and Exploited Children.

This translation in French was submitted by the authors and we reproduce it as supplied. It has not been peer reviewed. Our editorial processes have only been applied to the original abstract in English, which should serve as reference for this manuscript.

**Prise en charge clinique des abus sexuels pendant l'enfance : revue systématique et évaluation critique des recommandations de pratique en Europe**

**ABSTRACT:**

**Introduction** : La prise en charge des abus sexuels sur mineurs (ASM) nécessite des compétences spécialisées de la part des professionnels de la santé en raison de leur caractère très sensible, de leurs implications juridiques et de leurs graves effets sur la santé physique et mentale. Des recommandations de pratique clinique (RPC) standardisées et exhaustives peuvent avoir un rôle crucial dans cette prise en charge. Dans cette revue systématique, nous avons examiné les RPC nationales (RPCN) pour la prise en charge des ASM existants dans les pays européens afin d'évaluer leur qualité.

**Méthodes** : Nous avons systématiquement recherché des RPC dans six bases de données internationales et multiples sources de la littérature grise et rapporté nos résultats selon les recommandations PRISMA (*Preferred Reporting Items for Systematic Reviews and Meta-Analyses*). Les RPC éligibles devaient émaner d'agences sanitaires ou de sociétés savantes d’un des 34 pays européens du réseau COST Action 19106 (PERCA), et avoir été publiées entre janvier 2012 et novembre 2022. Deux chercheurs ont recherché, examiné, examiné et extrait les données indépendamment. La qualité du contenu des RPC incluses a été établie par comparaison aux RPC de référence de l’OMS publiées en 2017 et 2019. Nous avons utilisé l’outil AGREE II (*Appraisal of Guidelines for Research and Evaluation*) pour évaluer la qualité méthodologique et rédactionnelle des RPC évaluées.

**Résultats** : Parmi les 2 919 publications identifiées par les recherches dans les bases de données, aucune ne répondait aux critères d'inclusion. Sur 4 714 publications identifiées par d'autres méthodes, 24 RPCN provenant de 17 (50%) des PERCA ont été incluses. Dans 17 (50%) des PERCA, aucune RPCN n’a été trouvée. Le contenu des RPC variait considérablement entre chaque pays et au sein d’eux-mêmes. Les RPCN n’abordaient pas de nombreux éléments clefs présents RPC de référence de l’OMS, en particulier en matière de sécurité et d'évaluation des risques, d'interactions avec les soignants et d'interventions en santé mentale. L'évaluation par la grille AGREE II a révélé des faiblesses dans le développement des RCPG, en matière de rigueur scientifique, d'implication des parties prenantes, de mise en œuvre et d'évaluation.

**Interprétations** : Un nombre important de pays européens ne disposent pas d’une RPC pour la prise en charge des ASM, celles existantes ont une qualité très limitée. La réponse au défi de la prise en charge des ASC en Europe nécessite une approche coordonnée pour développer et mettre en œuvre des RPC de haute qualité. Nous plaidons pour le développement par une équipe multidisciplinaire de RPC paneuropéenne pour la prise en charge des ASC afin de garantir des soins de qualité aux victimes survivantes.

**Financement** : Un financement a été reçu par le Centre international pour les enfants disparus et exploités.

This translation in German was submitted by the authors and we reproduce it as supplied. It has not been peer reviewed. Our editorial processes have only been applied to the original abstract in English, which should serve as reference for this manuscript.

**Klinische Versorgung von sexuellem Missbrauch in der Kindheit: ein Systematic Review und kritische Bewertung von Leitlinien europäischer Länder**

**ABSTRACT**:

**Einleitung:** Der Umgang mit sexuellem Kindesmissbrauch (Child sexual abuse, CSA) verlangt von Angehörigen der Gesundheitsberufe (healthcare professionals, HCPs) aufgrund der Sensibilität des Themas, seiner rechtlichen Tragweite und seiner schwerwiegenden Auswirkungen auf die physische und psychische Gesundheit fachspezifische Fähigkeiten. Standardisierte, umfassende Leitlinien für die klinische Praxis (clinical practice guidelines , CPGs) können hierbei von entscheidender Bedeutung sein. Wir analysieren in dieser systematischen Übersichtsarbeit die vorhandenen nationalen Leitlinien europäischer Länder für die klinische Praxis (CSA national CPGs, NCPGs) und vergleichen sich in Bezug auf Qualität und Dokumentation.

**Methoden:** Es wurden sechs internationale Datenbanken und mehrere Quellen für graue Literatur systematisch von uns durchsucht, wobei die Berichterstattung nach den PRISMA-Standards (Preferred Reporting Items for Systematic Reviews and Meta-Analyses) erfolgte. Zum Einschluss in Frage kamen CSA-Leitlinien von nationalen Gesundheitsbehörden oder Fachgesellschaften aus 34 Netzwerkländern der COST-Action 19106 (COST Action 19106 Network Countries, CANC), die zwischen Januar 2012 und November 2022 veröffentlicht wurden. Je zwei unabhängige Forscher recherchierten, überprüften, untersuchten und extrahierten die Daten. Die NCPGs wurden mit den WHO-Leitlinien 2017 und 2019 auf ihre Vollständigkeit verglichen. Zur Beurteilung der Qualität und der Berichterstattung wurde das Appraisal of Guidelines for Research and Evaluation (AGREE II) angewandt.

**Ergebnisse:** Von den 2.919 Dokumenten, die bei der Datenbankrecherche ermittelt wurden, erfüllte keines die Einschlusskriterien. Von 4.714 Dokumenten, die mit anderen Methoden ermittelt wurden, wurden 24 NCPGs aus 17 (50 %) der CANC-Länder in unsere Bewertung einbezogen. In 17 (50 %) der in Frage kommenden Länder wurden keine NCPGs gefunden. Die Inhalte variierten sowohl innerhalb als auch zwischen den Ländern erheblich. Im Vergleich zu den WHO-Referenzstandards fehlten in den NCPGs zahlreiche Komponenten der state-of-the-art klinischen Praxis, dies betraf insbesondere die Bereiche Sicherheit und Risikobewertung, Interaktionen mit den Betreuungspersonen und psychische Gesundheitsmaßnahmen. Die Bewertung durch AGREE II ergab Mängel bei der Entwicklung der NCPGs in Bezug auf wissenschaftliche Genauigkeit, Einbeziehung von Interessengruppen sowie die Umsetzung und Bewertung.

**Interpretationen**: Eine beachtlichen Anzahl europäischer Länder hat keine NCPGs; die bestehenden NCPGs sind häufig unzureichend. Maßnahmen des Gesundheitswesens gegen CSA in Europa bedürfen eines koordinierten Ansatzes zur Entwicklung und Umsetzung hochwertiger Leitlinien. Wir sprechen uns dafür aus, dass ein multidisziplinäres Team eine gesamteuropäische CSA-Leitlinie entwickelt, um eine qualitativ hochwertige Versorgung der Überlebenden zu gewährleisten.

Finanzierung: Die Studie wurde durch das International Centre for Missing and Exploited Children finanziert.

This translation in Italian was submitted by the authors and we reproduce it as supplied. It has not been peer reviewed. Our editorial processes have only been applied to the original abstract in English, which should serve as reference for this manuscript.

**Trattamento del trauma da abuso sessuale in etá pediatrica: review sistematica e valutazione critica delle linee guida dei paesi europei**

ABSTRACT:

**Introduzione:** La gestione dell'abuso sessuale sui minori (child sexual abuse: CSA) richiede competenze specialistiche da parte degli operatori sanitari (healthcare professionals: HCP) a causa della sua delicatezza, delle implicazioni legali e dei gravi effetti sulla salute fisica e mentale. Linee guida standardizzate e complete per la pratica clinica (clinical practice guidelines: CPG) possono essere fondamentali. In questa revisione sistematica, abbiamo esaminato le CPG nazionali (national CPGs: NCPG) sui CSA esistenti nei Paesi europei per valutarne la qualità e la modalità di documentazione.

**Metodi:** Abbiamo effettuato una ricerca sistematica in sei banche dati internazionali e multiplo fonti di letteratura grigia secondo gli standard del Preferred Reporting Items for Systematic Reviews and Meta-Analyses (PRISMA). Le linee guida eleggibili erano linee guida CSA di agenzie o società sanitarie nazionali in 34 paesi della rete COST Action 19106 (COST Action 19106 Network Countries: CANC), pubblicate tra gennaio 2012 e novembre 2022. Due ricercatori indipendenti hanno cercato, esaminato, rivisto ed estratto i dati. Gli NCPG sono stati confrontati per completezza con le linee guida di riferimento dell’OMS 2017 e 2019. Abbiamo utilizzato l'Appraisal of Guidelines for Research and Evaluation (AGREE II) per valutarne la qualità e la modalità di documentazione.

**Risultati:** Su 2.919 documenti identificati dalla ricerca nei database, nessuno soddisfaceva i criteri di inclusione. Su 4.714 documenti identificati con altri metodi, sono stati inclusi 24 NCPG da 17 (50%) dei Paesi CANC. In 17 (50%) dei Paesi eleggibili non sono stati trovati NCPG. I documenti provenienti da paesi differenti così come quelli provenienti dallo stesso paese avevano contenuti significativamente differenti. Rispetto agli standard di riferimento dell'OMS, i NCPG erano privi di molti elementi caratteristici della pratica clinica di avanguardia, in particolare per quanto riguarda la sicurezza e la valutazione del rischio, le interazioni tra il personale dedito all’assistenza e gli interventi per la salute mentale. La valutazione di AGREE II ha rivelato carenze nello sviluppo degli NCPG per quanto riguarda il rispetto dei criteri scientifici, il coinvolgimento delle parti interessate, l'attuazione e la valutazione.

**Interpretazioni:** Un numero considerevole di Paesi europei non dispone di un NCPG; gli NCPG esistenti sono spesso insufficienti. La risposta sanitaria ai CSA in Europa richiede un approccio coordinato per sviluppare e migliorare la qualità delle CPG. Noi sosteniamo la necessità di un team multidisciplinare per sviluppare una linea guida paneuropea sul CSA per garantire un'assistenza di qualità ai pazienti.

**Finanziamenti:** Il finanziamento è stato fornito dal Centro internazionale per i bambini scomparsi e sfruttati.

This translation in Portuguese was submitted by the authors and we reproduce it as supplied. It has not been peer reviewed. Our editorial processes have only been applied to the original abstract in English, which should serve as reference for this manuscript.

**Cuidados clínicos no abuso sexual de crianças: uma revisão sistemática e avaliação crítica das normas de orientação clínica dos países europeus**

**Resumo:**

**Introdução:** A gestão clínica do abuso sexual de crianças (ASC) exige competências especializadas dos profissionais de saúde devido à sua sensibilidade, implicações legais e aos graves efeitos na saúde física e mental. Normas de orientação clínica (NOCs) padronizadas e abrangentes podem desempenhar um papel crucial nessa gestão. Nesta revisão sistemática, examinamos as NOC nacionais (NOCNs) existentes nos países europeus para avaliar a sua qualidade.

**Métodos:** Realizamos uma pesquisa sistemática em seis bases de dados internacionais e múltiplo fontes de literatura cinzenta e relatamos os nossos resultados de acordo com as orientações PRISMA (*Preferred Reporting Items for Systematic Reviews and Meta-Analyses*). As NOCs elegíveis deveriam ser provenientes de organismos de saúde ou sociedades científicas nacionais de um dos 34 países europeus da rede COST Action 19106 (CANC) e terem sido publicadas entre janeiro de 2012 e novembro de 2022. Dois investigadores conduziram, de forma independente, a pesquisa, revisão e extração de dados. A qualidade das NOCNs incluídas foi avaliada por comparação com as normas de referência da Organização Mundial da Saúde (OMS) de 2017 e 2019. Utilizamos a ferramenta AGREE II (*Appraisal of Guidelines for Research and Evaluation*) para avaliar a qualidade metodológica e de redação das NOCNs.

**Resultados:** Nenhum dos 2 919 registos identificados pela pesquisa nas bases de dados cumpriu aos critérios de inclusão. Dos 4 714 registos identificados por outros métodos, foram incluídas 24 NOCNs provenientes de 17 países (50%) da CANC. O conteúdo das NOCNs variou significativamente entre países e dentro de cada país. As NOCNs não abordaram muitos componentes presentes nas normas de referência da OMS para a prática clínica de ponta, especialmente no que diz respeito à segurança e avaliação de risco, interações com os cuidadores e intervenções em saúde mental. A avaliação através da AGREE II revelou limitações no desenvolvimento das NOCNs, em termos de rigor científico, envolvimento das partes interessadas, implementação e avaliação.

**Interpretações:** Um número significativo de países europeus não possui NOCNs, e as que existem frequentemente apresentam uma qualidade limitada. A resposta dos cuidados de saúde ao ASC na Europa exige uma abordagem coordenada para desenvolver e implementar NOCs de alta qualidade. Defendemos a criação de uma equipa multidisciplinar para desenvolver normas pan-europeias para o ASC, assegurando cuidados de qualidade aos sobreviventes.

**Financiamento:** Foi recebido financiamento do International Centre for Missing and Exploited Children.

This translation in Spanish was submitted by the authors and we reproduce it as supplied. It has not been peer reviewed. Our editorial processes have only been applied to the original abstract in English, which should serve as reference for this manuscript.

**La atención clínica del Abuso Sexual Infantil: revisión sistemática y evaluación de las guías de los países europeos**

**RESUMEN:**

**Introducción:** El manejo del Abuso Sexual Infantil (ASI) exige que los profesionales de la salud tengan unas habilidades especializadas, debido a la sensibilidad del tema, sus implicaciones legales y los graves efectos que tiene para la salud física y mental. Las guías para la práctica clínica (GPC) integrales y estandarizadas son fundamentales. En esta revisión sistemática, examinamos las GPC nacionales (GPCN) de ASI existentes en países europeos para evaluar su calidad y presentación de informes.

**Métodos:** Se ha hecho una búsqueda sistemática de seis bases de datos internacionales y varios recursos en la literatura gris, siguiendo los estándares marcados por Preferred Reporting Items for Systematic Reviews and Meta-Analyses (PRISMA). Las guías de ASI elegibles fueron las de agencias o sociedades nacionales de salud de 34 países de la red COST Action 19106 (CANC), publicadas entre enero de 2012 y noviembre de 2022. Dos personas investigadoras independientes buscaron, seleccionaron, revisaron y extrajeron los datos. Las GPCN fueron comparados en toda su integridad con las pautas de referencia de la OMS de 2017 y 2019. Se utilizaron las Appraisal of Guidelines for Research and Evaluation (AGREE II) para evaluar la calidad y la presentación de informes.

**Resultados:** De un total de 2.919 registros identificados en la búsqueda en las bases de datos, ninguno de ellos cumplió con los criterios de inclusión. De un total de 4.714 registros identificados a través de otros métodos, 24 GPCN de 17 (50%) de los países CANC fueron incluidos. En 17 (50%) países seleccionados en la red CANC no se pudieron encontrar GPCN. El contenido varió significativamente entre países. Los GPCN carecían de muchos componentes relacionados con la práctica clínica en comparación con los estándares de referencia de la OMS, particularmente cuando consideramos la evaluación de riesgos i protección, las interacciones con las personas cuidadoras, y las intervenciones en salud mental. La evaluación de AGREE II reveló deficiencias en el desarrollo de las GPCN en lo que respecta al rigor científico, la participación de las partes interesadas, y la implementación y evaluación.

**Interpretaciones:** Un numero notable de países europeos carecen de GPCN; y las guías existentes a menudo se quedan cortas. La respuesta sanitaria al ASI en Europa requiere un enfoque coordinado para desarrollar e implementar GPC de alta calidad. Abogamos por un equipo multidisciplinario para desarrollar una guía paneuropea de ASI para garantizar una atención de calidad para los supervivientes.

**Financiamiento:** Fue proporcionado por International Centre for Missing and Exploited Children.

This translation in Swedish was submitted by the authors and we reproduce it as supplied. It has not been peer reviewed. Our editorial processes have only been applied to the original abstract in English, which should serve as reference for this manuscript.

**Klinisk omhändertagande av sexuella övergrepp mot barn: en systematisk översikt med kritisk granskning av riktlinjer från europeiska länder**

**ABSTRAKT:**

**Bakgrund:** Omhändertagandet av sexuella övergrepp mot barn (SÖ) kräver specialiserad kompetens bland vårdpersonal på grund av ärendens känslighet, juridiska frågor och de allvarliga fysiska och psykiska hälsokonsekvenser som SÖ kan medföra. Standardiserade och heltäckande riktlinjer för klinisk praxis kan ha en viktig roll. I denna systematiska översikt granskade vi befintliga nationella riktlinjer för SÖ från europeiska länder och granskade deras kvalitet och innehåll.

**Metod:** Vi utförde ett systematiskt sök i sex internationella databaser och i flera grålitteratur databaser enligt PRISMA-standarderna (Preferred Reporting Items for Systematic Reviews and Meta-Analyses). Riktlinjer inkluderades om de var SÖ-riktlinjer utarbetade av nationella hälsovårdsmyndigheter eller andra organisationer i något av de 34 COST Action 19106 länderna (CANC) samt hade publicerats mellan januari 2012 och november 2022. Två oberoende forskare sökte, screenade, granskade och extraherade data. De nationella riktlinjerna jämfördes med referensriktlinjer från WHO (2017 och 2019). AGREE II (Appraisal of Guidelines for Research and Evaluation) användes för att bedöma kvalitet och rapportering.

**Resultat:** Av 2 919 sökresultat som identifierades genom databassökningar uppfyllde ingen inklusionskriterierna. Av 4 714 sökresultat som identifierats med andra metoder inkluderades 24 nationella riktlinjer från 17 (50 %) av CANC-länderna. I 17 (50 %) av de CANC länderna hittades inga nationella riktlinjer. Riktlinjernas innehåll varierade avsevärt inom och mellan länderna. Nationella riktlinjerna saknade många komponenter i state-of-the-art klinisk praxis jämfört med WHO:s referens, särskilt inom skydd och riskbedömning, samspel med omsorgspersoner och interventioner för psykisk hälsa. Bedömningen med AGREE II avslöjade brister i utvecklingen av nationella riktlinjer när det gäller vetenskaplig stringens, samverkan med intressenter samt implementering och utvärdering.

**Slutsatser**: Ett stort antal europeiska länder saknar en nationell riktlinje och befintliga nationella riktlinjer är ofta bristfälliga. Hälso- och sjukvårdens svar på SÖ i Europa kräver ett samordnat tillvägagångssätt för att utveckla och implementera kliniska riktlinjer av hög kvalitet. Vi förespråkar att ett multidisciplinärt team skulle utveckla en paneuropeisk SÖ-riktlinje för att säkerställa kvaliteten av vården för det som har blivit utsatta.

**Finansiering:** Studien finansierades av International Centre for Missing and Exploited Children.
