# Supplementary material for: Clinical care of childhood sexual abuse: a systematic review and critical appraisal of guidelines from European countries
Source: Lancet Reg Health Eur. 2024 Feb 21;39:100868. doi: 10.1016/j.lanepe.2024.100868 (PMC10899013; doi:10.1016/j.lanepe.2024.100868)
Supplement: Supplement [file mmc2.docx]

Table of Contents

[Supplement 1: Preliminary search strategy and results_CSA_Guidelines_MEDLINE_19December2021 1](#_Toc156924142)

[Supplement 2: COST Action 19106 network countries (CANC) 2020-2022 3](#_Toc156924143)

[Supplement 3: Search strategy in MEDLINE/EMBASE format 5](#_Toc156924144)

[Supplement 4: Grey literature search queries 8](#_Toc156924145)

[Supplement 5: Search strategy for other databases in free keyword format 9](#_Toc156924146)

[Supplement 6: Full-text records retrieved, reviewed, and excluded from the analysis (n=55) with reasons for exclusion by reference: published guidelines (n=23), scientific literature (n=17), and grey literature (n=15). 10](#_Toc156924147)

[Supplement 7: Data Template Extraction Sheet 15](#_Toc156924148)

[Supplement 8: Identified definitions of child sexual abuse from WHO 2017 and included national clinical practice guidelines (n=24) 31](#_Toc156924149)

[Supplement 9 - Figure 4: Distribution of standardized scores by domain for the 24 NCPGs 38](#_Toc156924150)

[Supplement 10 - Table 4: Child sexual abuse guidelines quality changes over the years from 2014-2017 and 2018-2022 39](#_Toc156924151)

# Supplement 1: Preliminary search strategy and results_CSA_Guidelines_MEDLINE_19December2021

| **Ovid MEDLINE(R) <1946 to December Week 2 2021>** | |  |
| --- | --- | --- |
| # | **Searches** | **Results** |
| 1 | Child abuse.mp. or Child Abuse/ | 35791 |
| 2 | Abused child.mp. | 285 |
| 3 | Child sexual abuse.mp. or Child Abuse, Sexual/ | 11000 |
| 4 | Child sexual exploitation.mp. | 91 |
| 5 | or/1-4 | 35983 |
| 6 | Diagnosis/ or Diagnosis, Differential/ or diagnosis.mp. | 3635005 |
| 7 | diagnostic workup.mp. | 5178 |
| 8 | investigation.mp. | 498924 |
| 9 | or/6-8 | 4052245 |
| 10 | (teen or teens or teanage*).tw. | 10098 |
| 11 | (adolesc* or preadolesc* or pre-adolesc* or juvenil*).tw. | 341530 |
| 12 | (youth or youths or youngster*).tw. | 71604 |
| 13 | ((young adj (person* or persons or people)) or early adult*).tw. | 35902 |
| 14 | (student or students or schoolchild*).tw. | 275275 |
| 15 | exp infant/ | 1198360 |
| 16 | exp Child/ | 2031446 |
| 17 | Young adult/ | 964570 |
| 18 | adolescent/ | 2143043 |
| 19 | (boy* or girl* or child or children or infant or infants or kid or kids).tw. | 1557496 |
| 20 | (pediatri* or paediatri*).tw. | 340969 |
| 21 | or/10-20 | 4657351 |
| 22 | Guideline Adherence/ or guidelines.mp. or Guideline/ or Practice Guideline/ | 442433 |
| 23 | clinical guideline.mp. | 1684 |
| 24 | recommendation.mp. | 41359 |
| 25 | recommendation*.mp. | 254034 |
| 26 | guideline*.mp. | 465241 |
| 27 | clinical gudeline*.mp. | 1 |
| 28 | or/22-27 | 646143 |
| 29 | **5 and 9 and 21 and 28** | **632** |
| 30 | **limit 29 to yr="2000-2022"** | **490** |

# Supplement 2: COST Action 19106 network countries (CANC) 2020-2022

| **No** | **Country** |
| --- | --- |
| 1 | Albania |
| 2 | Austria |
| 3 | Belgium |
| 4 | Bosnia and Herzegovina |
| 5 | Bulgaria |
| 6 | Croatia |
| 7 | Cyprus |
| 8 | Denmark |
| 9 | Estonia |
| 10 | Finland |
| 11 | France |
| 12 | Germany |
| 13 | Greece |
| 14 | Hungary |
| 15 | Iceland |
| 16 | Ireland |
| 17 | Israel |
| 18 | Italy |
| 19 | Latvia |
| 20 | Lithuania |
| 21 | Luxembourg* |
| 22 | Malta |
| 23 | Moldova |
| 24 | Netherlands |
| 25 | North Macedonia |
| 26 | Norway |
| 27 | Poland |
| 28 | Portugal |
| 29 | Romania |
| 30 | Slovenia |
| 31 | Spain |
| 32 | Sweden |
| 33 | Switzerland |
| 34 | Turkey |
| 35 | United Kingdom |

*Luxembourg joined the COST Action 19106 network in 2023 and was not included in the study.

# Supplement 3: Search strategy in MEDLINE/EMBASE format

1. Child abuse.mp. or Child Abuse/

2. Abused child.mp.

3. Child sexual abuse.mp. or Child Abuse, Sexual/

4. Child sexual exploitation.mp.

5. (sexual violence or sexual assault or rape or sex trafficking or sexual coercion).mp.

6. or/1-5

7. Diagnosis/ or Diagnosis, Differential/ or diagnosis.mp.

8. diagnostic workup.mp.

9. investigation.mp.

10. (management or management tool or medical management or assessment).mp.

11. (critical pathway or clinical pathway or care path or critical path or toolkit).mp.

12. or/7-11

13. (teen or teens or teenage*).mp.

14. (adolesc* or preadolesc* or pre-adolesc* or juvenil*).mp.

15. (youth or youths or youngster*).mp.

16. ((young adj (person* or persons or people)) or early adult*).mp.

17. (student or students or schoolchild*).mp.

18. exp infant/

19. exp Child/

20. Young adult/

21. adolescent/

22. (boy* or girl* or child or children or infant or infants or kid or kids).mp.

23. (pediatri* or paediatri*).mp

24. or/13-23

25. Guideline Adherence/ or guidelines.mp. or Guideline/ or Practice Guideline/

26. clinical guideline.mp.

27. recommendation.mp.

28. recommendation*.mp.

29. guideline*.mp.

30. clinical gudeline*.mp.

31. or/25-30

32. 6 and 12 and 24 and 31

33. limit 32 to yr="2012-2022"

# Supplement 4: Grey literature search queries

- child sexual abuse guidelines

-clinical guidance child sexual abuse violence diagnosis

-child adolescent abuse sex clinical guide Europe EU

-child sexual OR abuse exploitation OR assault rape sex trafficking sexual coercion diagnostic work up investigation management assessment clinical pathway care critical pathway infant child young adolescent teenager clinical guideline recommendation

# Supplement 5: Search strategy for other databases in free keyword format

(Child abuse or child sexual abuse or child sexual exploitation or sexual violence or sexual assault or rape or sex trafficking or sexual coercion)

AND (Diagnosis or diagnostic work up or investigation or management or assessment or clinical pathway or care path or critical pathway)

AND (Infant or child or young adult or adolescent or teenager or boy or girl)

AND (Clinical guideline or guideline or practice guideline or recommendation)

# Supplement 6: Full-text records retrieved, reviewed, and excluded from the analysis (n=55) with reasons for exclusion by reference: published guidelines (n=23), scientific literature (n=17), and grey literature (n=15).

| **NCPGs** | **Country** | **Reasons for exclusion** |
| --- | --- | --- |
| **^1^** | Austria | Institutional document |
| **^2^** | Austria | Not CPG - Institutional form |
| **^3^** | Austria | Not CPG - Institutional form |
| **^4^** | Belgium | Not CPG – Institutional forms |
| **^5^** | Belgium | Does not specifically address HCP care |
| **^6^** | Croatia | Duplicate HCP protocol to 2014 included report |
| **^7^** | Finland | Book chapter |
| **^8^** | Finland | Book chapter |
| **^9^** | France | Does not address medical management of child victims (is perpetrator-focused) |
| **^10^** | France | Outdated |
| **^11^** | Greece | Incomplete CPG |
| **^12^** | Italy | Regional guideline |
| **^13^** | Italy | Early date |
| **^14^** | Malta | No reasons |
| **^15^** | Portugal | Early date |
| **^16^** | Portugal | Not national, not CPG |
| **^17^** | Portugal | Targets church staff; does not address HCPs |
| **^18^** | Portugal | Does not address HCPs |
| **^19^** | Portugal | Does not address children; does not provide specific advice for HCPs |
| **^20^** | Portugal | Incomplete CPG |
| **^21^** | Slovenia | Does not address HCP clinical management |
| **^22^** | Sweden | Not CPG – Instructions forensic collection |
| **^23^** | Sweden | A checklist |

| Scientific literature | Excluded |
| --- | --- |
| [^24^](#_ENREF_24) | Not CPG |
| [^25^](#_ENREF_25) | Not CPG |
| [^26^](#_ENREF_26) | Not CPG |
| [^27^](#_ENREF_27) | Not CPG |
| [^28^](#_ENREF_28) | Not CPG |
| [^29^](#_ENREF_29) | Not CPG |
| [^30^](#_ENREF_30) | Not CPG |
| [^31^](#_ENREF_31) | Not CPG |
| [^32^](#_ENREF_32) | Not CPG |
| [^33^](#_ENREF_33) | Not CPG |
| [^34^](#_ENREF_34) | Not CPG |
| [^35^](#_ENREF_35) | Not CPG |
| [^36^](#_ENREF_36) | Not CPG - systematic review |
| [^37^](#_ENREF_37) | Not CPG - systematic review |
| [^38^](#_ENREF_38) | Not CPG - systematic review |
| [^39^](#_ENREF_39) | Not CPG |
| [^40^](#_ENREF_40) | Not CPG |

| Grey literature | Country | Reasons for exclusion |
| --- | --- | --- |
| [^41^](#_ENREF_41) | Albania | Qualitative research |
| [^42^](#_ENREF_42) | Bulgaria | National policy framework |
| [^43^](#_ENREF_43) | Cyprus | National Strategy and Action Plan |
| [^44^](#_ENREF_44) | Denmark | Cohort study |
| [^45^](#_ENREF_45) | Denmark | Policy paper for safeguarding children |
| [^46^](#_ENREF_46) | Finland | Not CPG |
| [^47^](#_ENREF_47) | Finland | Safeguarding policy and training material for school employees and students |
| [^48^](#_ENREF_48) | Germany | National report |
| [^49^](#_ENREF_49) | Greece | European project: Report on current knowledge and practices on Child institutional abuse |
| [^50^](#_ENREF_50) | Netherlands | Not CPG |
| [^51^](#_ENREF_51) | Netherlands | Child protection policy |
| [^52^](#_ENREF_52) | Romania | Report on trafficking, prostitution etc. |
| [^53^](#_ENREF_53) | Switzerland | European Project: Report on child abuse and practices |
| [^54^](#_ENREF_54) | UK | Guidance on child house protection |
| [^55^](#_ENREF_55) | UK | A guide for those working with children who have or may have been sexually abused |

1. E. M-T. FOKUS: Pilot project "Suspicion of sexual offence”. Austria; 2020.

2. AKH_Wein. FOKUS: Module - Sexually Transmitted Infections (STI). Austria: University Hospital for Paediatrics and Jewish Medicine; 2021.

3. AKH_Wein. FOKUS: Module - Suspected Child Sexual Abuse. Austria: University Hospital for Paediatrics and Jewish Medicine; 2021.

4. Keygnaert I., Wuestenbergs J., Decorte R., Deforce D., Van Hoofstat D., Marcotte A., et al. Step-by-step plan for forensic investigation in a care center after sexual violence (minors). In The Institute for the Equality of Women and Men. Belgium 2020.

5. Irm Vinck, Wendy Christiaens, Pascale Jonckheer, Geneviève Veereman, Laurence Kohn, Nicole Dekker, et al. How To Improve The Detection Of Child Abuse In Belgium. Belgium: KCE; 2016.

6. Soba Z. Protocol om procedure in the case of sexual violence. Croatia, Zagreb; 2018.

7. Leena Laitinen, Eeva Nikkola, Mäkelä A. Sexual violence. Finland.

8. Nikkola E, Tupola S. Child abuse. Finland

9. Baron-Laforet S. Support for perpetrators of sexual assault against minors under the age of 15. France; 2009.

10. Gilbert V. Identification and reporting of incest by doctors: recognizing intrafamilial sexual abuse in minors. France: Haute Autorité de Santé; 2011.

11. Soldatou A, Michala L. Sexual abuse. Greece.

12. Savino C, Berlingerio I, Grazia M, Barbaro F. Sexual violence against minors. A multidisciplinary approach. Italy: Giovanni XXIII Pediatric Hospital AOU Policlinico-Giovanni XXIII; 2020.

13. Giolito MR. Sexual abuse in children prepuber, Requirements and recommendations for appropriate evaluation. Rome, Italy; 2010.

14. Mariella M. Child Protection Policy. Malta: Mater Dei Hospital; 2022.

15. Magalhães T, Ribeiro CS, Jardim P, ,, Vieira DN. Forensic Procedures - Physical and Collection of Traces in Children and Young People - In the Scope of Information Collection, Examination Victims of Physical and/or Sexual Abuse. Acta Med Port. 2011; 24(2): :339-48.

16. Martins MM, de Macedo MS, Mira R. Pocket Guide to Sexual Violence – for Professionals. Portugal; 2015.

17. Sotto-Mayor A, Fernandes C, Perloiro F. Manual SPC System Of Protection And Beware Juvenile And Adults Vulnerable. Lisbon; 2018.

18. APAV. CARE Manual - Support for children and young people who are victims of sexual violence. Lisbon; 2019.

19. Silva D, Mendes, Lia , Mendes T. Manual of good practices for professionals in the care of victims of sexual violence. Portugal; 2021.

20. Mendes P, Maxisaúde U. Suspected sexual abuse in children: how to act. Portugal; 2013.

21. CoE. National Guidelines for Barnahus in Slovenia. Slovenia; 2019.

22. NCK. Guide for care after sexual assaul. Sweden; 2022.

23. BLF_LPO. Suspected sexual abuse of children - Checklist for medical examination, trace assurance and cooperation. Sweden: BLF, Region Skåne's child protection team and LPO child and youth health; 2022.

24. Hoffmann U, Fegert JM, Konig E, Maier A, Herberhold M. Development of safeguarding measures against (sexual) abuse in the medical-therapeutic field. Kindheit und Entwicklung: Zeitschrift fur Klinische Kinderpsychologie. 2021;30(4):227-35.

25. Hornor G. Child Maltreatment Prevention: Essentials for the Pediatric Nurse Practitioner. Journal of Pediatric Health Care. 2022;36(2):193-201.

26. Amin A, MacMillan H, Garcia-Moreno C. Responding to children and adolescents who have been sexually abused: WHO recommendations. Paediatr Int Child Health. 2018;38(2):85-6.

27. Born M, Schwier F, Stoever B, Mentzel HJ, Freiberg J. The German Evidence-Based Child Protection Guideline - Imaging in Suspected Child Abuse. ROFO Fortschr Geb Rontgenstr Nuklearmed. 2020;192(4):343-8.

28. Capri P. The expert evaluation of the child in case of sexual abuse. Maltrattamento e Abuso All'Infanzia: Rivista Interdisciplinare. 2016;18(2):13-29.

29. de Santé HA. Maltraitance chez l’enfant: repérage et conduite à tenir. 2017.

30. Repérage H. signalement de l’inceste par les médecins: reconnaître les maltraitances sexuelles intrafamiliales chez le mineur. Recommandations HAS. 2011.

31. Fegert JM, Hoffmann U. Kinderschutz und Schutzkonzepte im klinischen und ambulanten Bereich. Kindh Entwickl. 2021;30(4):205-7.

32. Vrolijk-Bosschaart TF, Nagtegaal M, Brilleslijper-Kater SN, Benninga MA, Lindauer RJL, Teeuw AHR. How do you recognise sexual abuse in children?. [Dutch]. Ned Tijdschr Geneeskd. 2018;162(no pagination).

33. Richter P, Bartels MB, Kieslich M. Diagnostics and Significance Evaluation in Cases of Suspected Child Sexual Assault An Analysis of 210 Cases. Klinische Padiatr. 2021;233(05):237-45.

34. Fegert JM, Hoffmann U, Sprober N, Liebhardt H. Child sexual abuse: Epidemiology, clinical diagnostics, therapy, and prevention. [German]. Bundesgesundheitsblatt - Gesundheitsforschung - Gesundheitsschutz. 2013;56(2):199-207.

35. Ayou C, Vabres N, Poullaouec C, Lemesle M, Gras-le Guen C, Launay E, et al. [Social or judicial alert in child abuse: Announcement guidelines and simulation in child welfare training with standardized patients]. Encephale. 2022;48 Suppl 1:S34-S8.

36. Brunton R, Dryer R. Child Sexual Abuse and Pregnancy: A Systematic Review of the Literature. Child Abuse Negl. 2021;111:104802.

37. Chen CJ, Chen YW, Chang HY, Feng JY. Screening Tools for Child Abuse Used by Healthcare Providers: A Systematic Review. J Nurs Res. 2022;30(1):e193.

38. Rahnavardi M, Shahali S, Montazeri A, Ahmadi F. Health care providers’ responses to sexually abused children and adolescents: a systematic review. BMC Health Services Research. 2022;22(1):1-11.

39. Ashby J, Rogstad K, Forsyth S, Wilkinson D. Spotting the Signs: a national toolkit to help identify young people at risk of child sexual exploitation. Sex Transm Infect. 2015;91(4):231.

40. Barbero E, Rossi C, Poletti E, Mazza S, Fuser L, Visentin R, et al. [Sexual abuse in a child or adolescent: the first approach in the Emergency Room]. [Italian]. La Pediatria medica e chirurgica : Medical and surgical pediatrics. 2013;35(3):141-8.

41. Burazeri G, QirjakoÇela G, Tahsini I. Child Sexual Abuse in the Circle of Trust in Albania - A Qualitative Research on the Perspectives and Perceptions of Professionals, Parents and Children: Terre des hommes Albania; 2015 [Available from: <https://childhub.org/sites/default/files/library/attachments/RAPORTI%20CSA_Eng%20%28print%29.pdf>.

42. CoE. National Programme for Prevention of Violence and Abuse of Children 2017-2020, Bulgaria Bulgaria2017 [Available from: <https://www.coe.int/en/web/children/resource-centre/-/asset_publisher/lCGym75EBhwG/content/national-programme-for-prevention-of-violence-and-abuse-of-children-2017-2020-bulgaria?inheritRedirect=false&redirect=https%3A%2F%2Fwww.coe.int%2Fweb%2Fchildren%2Fresource-centre%3Fp_p_id%3D101_INSTANCE_lCGym75EBhwG%26p_p_lifecycle%3D0%26p_p_state%3Dnormal%26p_p_mode%3Dview%26p_p_col_id%3Dcolumn-6%26p_p_col_pos%3D1%26p_p_col_count%3D2>.

43. Cyprus. Cyprus: Deputy Ministry of Social Welfare Social Welfare Services; 2022 [Available from: <http://www.dmsw.gov.cy/dmsw/sws.nsf/home_el/home_el?opendocument>.

44. Elklit A. Treatment of Danish Survivors of Child Sexual Abuse-A Cohort Study. Behavioral sciences (Basel, Switzerland). 2015;5(4):589-601.

45. ADRA. ADRA Denmark’s Child Safeguarding Policy 2021 [Available from: <https://www.adra.dk/wp-content/uploads/2021/03/ADRA-Denmark-Child-Safeguarding-Policy-updated-2021.pdf>.

46. Piha J, Aronen E, Joki-Erkkilä M, Komulainen J, Korkman J, Raipela J, et al. [Update in Current Care guidelines. Evaluation of a suspected child sexual abuse]. Duodecim. 2013;129(12):1290-1.

47. ISH. Child Safeguarding - Prevention Protection Support - Policy and Procedures for ISH Handbook 2019 [Available from: <https://ishelsinki.fi/wp-content/uploads/2019/08/ISH_Child-Safeguarding_A4_web.pdf>.

48. Galm B, Derr R. Combating Child Abuse and Neglect Child Protection in Germany - National Report: Deutsches Jugendinstitut; [

49. George N, Αggelika G, Georgia P, Maria P. The childinstitutional abuse:current knowledge and practices - GreeceReport Greece: Institut of Child Health – Department of Mental Health and Social Welfare; 2017 [Available from: <http://www.sasca.eu/wp-content/uploads/2018/03/National-report-Greece.pdf>.

50. Vrolijk-Bosschaart TF, Brilleslijper-Kater SN, Benninga MA, Lindauer RJL, Teeuw AH. Clinical practice: recognizing child sexual abuse-what makes it so difficult? European journal of pediatrics. 2018;177(9):1343-50.

51. DCI-ECPAT. Child Protection Policy - Defence for Children International Netherlands – ECPAT Netherland Netherlands: Defence for Children International – ECPAT Nederland; 2016 [Available from: <https://defenceforchildren.nl/media/1281/child-protection-policy-complete-version.pdf>.

52. ECPAT. Execitive Summary - Romania [Available from: <https://ecpat.org/wp-content/uploads/2021/08/EXSUM_A4A_EU_ROMANIA-1.pdf>.

53. Jud A, Mirjam S. CAN surveillance in Switzerland: current policies and practices Switzerland: Lucerne University of Applied Sciences and Arts; 2014 [Available from: <http://can-via-mds.eu/sites/default/files/WS.1_D4.8_CAN%20Surveillance%20in%20Switzerland_Current%20Policies%20and%20Practices.pdf>.

54. Gov.UK. Child House: local partnerships guidance (accessible version) UK: <https://www.gov.uk/>; 2021 [Available from: <https://www.gov.uk/government/publications/child-sexual-abuse-child-house/child-house-local-partnerships-guidance-accessible-version>.

55. Sabin N. A guide for those working with children who have or may have been sexually abused UK: Centre of expertise on child sexual abuse; 2022 [Available from: <https://childhub.org/sites/default/files/library/attachments/Communicating_with_children_english.pdf>.

# Supplement 7: Data Template Extraction Sheet

|  |  | **Name of Guideline Under Review:** |  |  |  |
| --- | --- | --- | --- | --- | --- |
|  |  | **Year of guideline publication:** |  |  |  |
|  |  | **Country Origin of Guideline:** |  |  |  |
|  |  | **Reviewer Name:** |  |  |  |
|  |  | **Date of review:** |  |  |  |
|  |  | **Version reviewed (e.g., native language vs Google vs. another translated version):** |  |  |  |
|  |  | **General comments about guideline related to strengths/gaps (ex., 'Does not address CSA of boys'; OR 'focuses on women with only single chapter on minors'; OR 'very well organized and information is easily accessible')** |  |  |  |
|  | **Item #** | **W.H.O. 2017 Guidelines** | **Score: Absent; Partially Present; Present** | **Page number(s) with verbatim quote** | **Comments** |
|  | **A. CHILD- OR ADOLESCENT-CENTRED CARE/FIRST-LINE SUPPORT** | |  |  |  |
| **GP 1** |  | **GP1** Health-care providers should provide first-line support that is gender sensitive and child or adolescent centred, in response to disclosure of sexual abuse. This includes: |  |  |  |
|  | **1** | ·       listening respectfully and empathetically to the information that is provided; |  |  |  |
|  | **2** | ·       inquiring about the child’s or adolescent’s worries or concerns and needs, and answering all questions; |  |  |  |
|  | **3** | ·       offering a non-judgmental and validating response; |  |  |  |
|  | **4** | ·       taking actions to enhance their safety and minimize harms, including those of disclosure and, where possible, the likelihood of the abuse continuing, this includes ensuring visual and auditory privacy; |  |  |  |
|  | **5** | ·       providing emotional and practical support by facilitating access to psychosocial services; |  |  |  |
|  | **6** | ·       providing age-appropriate information about what will be done to provide them with care, including whether their disclosure of abuse will need to be reported to relevant designated authorities; |  |  |  |
|  | **7** | ·       attending to them in a timely way and in accordance with their needs and wishes; |  |  |  |
|  | **8** | ·       prioritizing immediate medical needs and first-line support; |  |  |  |
|  | **9** | ·       making the environment and manner in which care is being provided appropriate to age, as well as sensitive to the needs of those facing discrimination related to, for example, disability or sexual orientation; |  |  |  |
|  | **10** | ·       minimizing the need for the them to go to multiple points of care within the health facility; |  |  |  |
|  | **11** | ·       empowering non-offending caregivers with information to understand possible symptoms and behaviours that the child or adolescent may show in the coming days or months and when to seek further help. |  |  |  |
|  | **B. MEDICAL HISTORY, PHYSICAL EXAMINATION AND DOCUMENTATION OF FINDINGS** | |  |  |  |
| **GP2** |  | In line with the principle of “do no harm”, when the medical history is being obtained and, if needed, a forensic interview is being conducted, health-care providers should seek to minimize additional trauma and distress for children and adolescents who disclose sexual abuse. This includes: |  |  |  |
|  | **12** | ·       minimizing need to repeatedly tell their history; |  |  |  |
|  | **13** | ·       interviewing them on their own (i.e. separately from their caregivers), while offering to have another adult present as support; |  |  |  |
|  | **14** | ·       building trust and rapport by asking about neutral topics first; |  |  |  |
|  | **15** | ·       conducting a comprehensive assessment of their physical and emotional health, in order to facilitate appropriate decisions for conducting examinations and investigations, assessing injuries and providing treatment and/or referrals; |  |  |  |
|  | **16** | ·       asking clear, open-ended questions without repetitions; |  |  |  |
|  | **17** | ·       using language and terminology that is appropriate to age and non-stigmatizing; |  |  |  |
|  | **18** | ·       allowing the child or adolescent to respond in the manner of their choice, including, for example, by writing, drawing or illustrating with models. |  |  |  |
| **GP3** |  | In conducting physical examinations and, where needed, forensic investigations, health-care providers should seek to minimize additional harms, trauma, fear and distress, and respect the autonomy and wishes of children or adolescents. This includes: |  |  |  |
|  | **19** | ·       maximizing efforts to have them undergo only one examination; |  |  |  |
|  | **20** | ·       offering information about the implications of positive or negative findings; |  |  |  |
|  | **21** | ·       minimizing delays while conducting the examination in accordance with the child’s or adolescent’s wishes; |  |  |  |
|  | **22** | ·       explaining what will be done, prior to each step; |  |  |  |
|  | **23** | ·       offering choice in the sex of the examiner, where possible; |  |  |  |
|  | **24** | ·       making sure there is another adult present during the examination; |  |  |  |
|  | **25** | ·       using age-appropriate visual aids and terms to explain the examination procedures; |  |  |  |
|  | **26** | ·       using examination instruments and positions that minimize physical discomfort and psychological distress; |  |  |  |
|  | **27** | ·       ensuring collection of forensic evidence is based on the account of the abuse and on what evidence can be collected, stored and analysed; |  |  |  |
|  | **28** | ·       not conducting virginity testing (two-finger test or per-vaginal examination), as it increases distress and does not indicate whether or not abuse took place; |  |  |  |
|  | **29** | ·       not routinely using speculums, anoscopes and digital or bimanual examinations of the vagina or rectum of pre-pubertal children, unless medically indicated; if they are used, sedation or general anaesthesia should be considered. |  |  |  |
| **GP4** |  | Health-care providers should accurately and completely document findings of the medical history, physical examination and forensic tests and any other relevant information, for the purposes of appropriate followup and supporting survivors in accessing police and legal services, while at the same time protecting confidentiality and minimizing distress for children or adolescents and their caregivers. This includes: |  |  |  |
|  | **30** | ·       using a structured format for recording the findings; |  |  |  |
|  | **31** | ·       recording verbatim statements for accurate and complete documentation; |  |  |  |
|  | **32** | ·       noting down discrepancies between the child´s or adolescent´s and the caregivers´ account, if any, without interpretation; |  |  |  |
|  | **33** | ·       recording a detailed and accurate description of the symptoms and injuries; |  |  |  |
|  | **34** | ·       where no physical evidence is found, noting that absence of physical evidence does not mean that abuse did not occur; |  |  |  |
|  | **35** | ·       documenting the child’s or adolescent’s emotional state, while noting that no particular state is indicative of sexual abuse; |  |  |  |
|  | **36** | ·       seeking informed consent, as appropriate, for taking any photographs and/or videos, after explaining how they will be used. |  |  |  |
|  | **36a** | ·       handling all collected information confidentially. |  |  |  |
|  | **C. HIV POST-EXPOSURE PROPHYLAXIS TREATMENT AND ADHERENCE** | |  |  |  |
| **R1** | **37** | ·       HIV post-exposure prophylaxis (PEP) should be offered, as appropriate, to children and adolescents who have been raped involving oral, vaginal or anal penetration with a penis, and who present within 72 hours of the incident. |  |  |  |
| **R2** | **38** | ·       A 28-day prescription of antiretroviral drugs (ARVs) should be provided for HIV PEP, following initial risk assessment. |  |  |  |
| **R3** | **39** | ·       A triple-therapy regimen (i.e. with three drugs) of ARVs is preferred but a two-drug regimen is also effective. |  |  |  |
| **R4** | **40** | ·       Adherence counselling should be an important element in the provision of HIV PEP to survivors of sexual assault or rape. |  |  |  |
|  | **D. PREGNANCY PREVENTION AND MANAGEMENT AMONG GIRLS WHO HAVE BEEN SEXUALLY ABUSED** | | |  |  |
| **R5** | **41** | ·       Offer emergency contraception to girls who have been raped involving peno-vaginal penetration and who present within 120 hours (5 days) of the incident. |  |  |  |
| **GP5** | **42** | ·       If a girl is pregnant as a result of the rape, she should be offered safe abortion to the full extent of the law. |  |  |  |
|  | **E. POST-EXPOSURE PROPHYLAXIS FOR CURABLE AND VACCINE-PREVENTABLE SEXUALLY TRANSMITTED INFECTIONS** | | |  |  |
| **R6** | **43** | ·       Presumptive (or prophylactic) treatment for gonorrhoea, chlamydia and syphilis is suggested for children and adolescents who have been sexually abused involving oral, genital or anal contact with a penis, or oral sex, particularly in settings where laboratory testing is not feasible. |  |  |  |
| **R7** | **44** | ·       For children and adolescents who have been sexually abused and who present with clinical symptoms, syndromic case management is suggested for vaginal/urethral discharge (gonorrhoea, chlamydia, trichomoniasis), and for genital ulcers (herpes simplex virus, syphilis and chancroid), particularly in settings where laboratory testing is not feasible. |  |  |  |
| **R8** | **45** | ·       Hepatitis B vaccination without hepatitis B immunoglobulin should be offered, as per national guidance. |  |  |  |
| **R9** | **46** | ·       Human papillomavirus vaccination should be offered to girls in the age group 9–14 years, as per national guidance. |  |  |  |
|  | **F. PSYCHOLOGICAL AND MENTAL HEALTH INTERVENTIONS IN THE SHORT TERM AND LONGER TERM** | | |  |  |
| **GP6** | **47** | ·       For children and adolescents who have recently been sexually abused, and who experience symptoms of acute traumatic stress (within the first month), health-care providers should offer/continue to offer first-line support that is gender sensitive and child or adolescent centred, as described in Good Practice Statement 1. |  |  |  |
| **R10** | **48** | ·       Psychological debriefing should not be used in an attempt to reduce the risk of post-traumatic stress, anxiety or depressive symptoms. |  |  |  |
| **R11** | **49** | ·       Cognitive behavioural therapy (CBT) with a trauma focus should be considered for children and adolescents who have been sexually abused and are experiencing symptoms of post-traumatic stress disorder (PTSD). |  |  |  |
| **R12** | **50** | ·       When safe and appropriate to involve at least one nonoffending caregiver, CBT with a trauma focus should be considered for both: (i) children and adolescents who have been sexually abused and are experiencing symptoms of PTSD; and (ii) their non-offending caregiver(s). |  |  |  |
| **R13** | **51** | ·       Psychological interventions, such as CBT, may be offered to children and adolescents with behavioural disorders, and caregiver skills training to their non-offending caregivers. |  |  |  |
| **R14** | **52** | ·       Psychological interventions, such as CBT and interpersonal psychotherapy (IPT) may be offered to children and adolescents with emotional disorders, and caregiver skills training to their non-offending caregivers. |  |  |  |
|  | **G. ETHICAL PRINCIPLES AND HUMAN RIGHTS STANDARDS FOR REPORTING CHILD OR ADOLESCENT SEXUAL ABUSE** | | |  |  |
| **GP7** |  | Whether health-care providers have to comply with a legal or policy requirement, or they are guided by an ethical duty to report known or suspected cases of child or adolescent sexual abuse, they should balance the need to take into account the best interests of that child or adolescent with their evolving capacities to make autonomous decisions. This includes: |  |  |  |
|  | **53** | ·       assessing the implications of reporting for their health and safety and taking steps to promote their safety; |  |  |  |
|  | **54** | ·       protecting their privacy (for example, in dealing with the media); |  |  |  |
|  | **55** | ·       promoting their health, by providing immediate medical care and first-line support; |  |  |  |
|  | **56** | ·       providing information about the obligation to report and limits of confidentiality; |  |  |  |
|  | **57** | ·       documenting the reporting process and maintaining confidentiality of the documented information. |  |  |  |
|  | **58**  **(Not scored)** | Health managers and policymakers should: |  |  |  |
|  | **59** | ·       be aware of any legal requirements to report known or suspected cases of abuse; |  |  |  |
|  | **60** | ·       facilitate health-care providers to receive training on when and how to report; |  |  |  |
|  | **61** | ·       address health-care providers’ beliefs and values that can adversely affect their reporting practices; |  |  |  |
|  | **62** | ·       establish systems and policies for record-keeping and information sharing that protect confidentiality; |  |  |  |
|  | **63** | ·       work with other agencies or institutions, including child protection and police services, in order to coordinate an appropriate response. |  |  |  |
|  | **64**  **(Not scored)** | Actions that are not in line with the principle of evolving capacities include: |  |  |  |
|  | **65** | ·       reporting consensual sexual activity between adolescents, unless that adolescent’s safety is at risk; |  |  |  |
|  | **66** | ·       informing parents/caregivers where adolescents, depending on their age and maturity, express their preference not to involve their parents/caregivers, unless the adolescent’s safety is at risk. |  |  |  |
|  |  |  |  |  |  |
|  |  |  |  |  |  |
|  | **W.H.O. 2019 Guideline** | |  |  |  |
|  | **A. INITIAL IDENTIFICATION** | |  |  |  |
| **R1** | **67** | Health care providers should be alert to the clinical features associated with child maltreatment and associated risk factors and assess for child maltreatment without putting the child at increased risk. |  |  |  |
| **R2** | **68** | Health care providers should not use a universal screening approach (e.g. a standard instrument, set of criteria, or questions asked of all children in health care encounters) to identify possible child maltreatment. |  |  |  |
| **R3** | **69** | Health care providers should consider exposure to child maltreatment when assessing children with conditions that may be caused or complicated by maltreatment (see Boxes 1-6: Examples of clinical conditions associated with maltreatment and alerting features), in order to improve diagnosis/identification and subsequent care, without putting the child at increased risk. |  |  |  |
| **R4** | **70** | Written information on child maltreatment should be available in health-care settings in the form of posters, and pamphlets or leaflets *(with appropriate warnings about taking them home in case that could compromise safety).* |  |  |  |
| **GP1** |  | Health-care providers should seek explanations for any injuries or symptoms that may be caused by physical, sexual, emotional abuse or neglect from both the parent and the carer, and the child or young person in an open and non-judgemental manner. |  |  |  |
|  |  | Health care providers should: |  |  |  |
|  | **71** | **Be alert** for an implausible, inadequate or inconsistent explanation for any of the alerting features (see lists below). All of them can be a sign for child maltreatment – however none of them provides sufficient proof for the occurrence of child maltreatment.  ALERTING FEATURES: Sexual abuse  Anogenital signs and symptoms:  • A genital, anal or perianal injury (e.g. bruising, laceration, swelling or abrasion)  • A persistent or recurrent genital or anal symptom (for example, bleeding, dysuria or discharge) that is associated with behavioural or emotional change and that has no medical explanation.  • Foreign bodies in the vagina or anus. (Foreign bodies in the vagina may be indicated by offensive vaginal discharge).  Sexually transmitted infections:  • Including symptoms in the mouths or rarely in infected joints (gonorrhoeal septic arthritis).  Pregnancy in a child or young teen  Sexualised behaviour   ALERTING FEATURES: Any form of maltreatment may be associated with  • Marked change in behaviour or emotional state  • Recurrent nightmares containing similar themes  • Extreme distress  • Markedly oppositional behaviour  • Withdrawal of communication  • Withdrawn  Some of these features may also be seen in a wide range of adolescents for other reasons, including use of drugs. |  |  |  |
|  | **72** | ·       **Consider** child maltreatment when maltreatment is one possible explanation for the alerting feature or is included in the differential diagnosis. |  |  |  |
|  | **73** | ·       **Suspect** child maltreatment when there is a serious level of concern about the possibility of child maltreatment. |  |  |  |
|  | **74** | ·       **Exclude** maltreatment when a suitable explanation is found for alerting features. |  |  |  |
|  | **C. SAFETY AND RISK ASSESSMENT** | |  |  |  |
| **GP6** |  | Promoting and protecting the physical and emotional safety of the child or adolescent must be the primary consideration throughout the course of care. This means that, with the participation from the child and adolescent (and their non-offending caregivers, as appropriate,) health-care providers need to consider all potential harms and take actions that will minimize the negative consequences for the child or adolescent, including the likelihood of the maltreatment continuing. |  |  |  |
|  |  | Assessing safety and developing a safety plan for children and non-offending caregivers includes: |  |  |  |
|  | **75** | •                     assessing the child or adolescent’s physical and emotional safety needs; |  |  |  |
|  | **76** | •                     involving the child and caregivers in safety planning, where safe to do so, prioritizing the physical and emotional wellbeing of the child or adolescent; |  |  |  |
|  | **77** | •                     considering the risk of recurrence of child maltreatment taking into account whether the perpetrator of sexual or physical abuse has access to the child; whether caregivers are able to protect the child, and whether the child feels safe to return home; |  |  |  |
|  | **78** | •                     considering that different types of violence, and especially child maltreatment and intimate partner violence, often co-occur in the same household and that spouses, siblings and other members of the household might also be at risk of violence; |  |  |  |
|  | **79** | •                     involving other relevant agencies, in consultation with the child or adolescent, if the child’s safety is at risk. Information including contact details of relevant agencies should be made available to health care providers. In some settings no legal mechanism may be available to separate children from perpetrators of maltreatment in their current living arrangements or removing the child or adolescent may expose them to an even less safe environment. In such situations careful and frequent follow-up by health workers will be particularly important. |  |  |  |
|  | **80** | •                     always following up on all referrals; |  |  |  |
|  | **81** | •                     making a plan for follow-up contact with the child and/or caregivers, including what will happen if the child cannot be reached. |  |  |  |
|  |  |  |  |  |  |
|  |  | If assessment instruments are used to determine risk: |  |  |  |
|  | **82** | •                     be aware of the many factors that influence the risk of recurrence that may not be accounted for by assessment instruments; |  |  |  |
|  | **83** | •                     treat instruments as a tool to enhance or expand clinical judgement, not as a substitute for clinical judgement. |  |  |  |
|  | **D. INTERACTING WITH CAREGIVERS** | |  |  |  |
| **GP7** |  | The interaction with caregivers when child maltreatment is suspected is complicated by the fact that the caregiver might be the perpetrator or may have allowed the maltreatment or felt powerless to stop it or may also be victim of violence in the home. Good interaction will influence outcomes for and safety of the child or adolescent. When interacting with caregivers when child maltreatment is suspected, health care providers should therefore: |  |  |  |
|  | **84** | • consider that the caregiver might be the perpetrator or, in the case of a non-offending caregiver may have allowed the maltreatment to continue or felt powerless to stop it, and being aware of potential safety concerns for the child; |  |  |  |
|  | **85** | • consider that the caregiver might also be affected by violence in the home; |  |  |  |
|  | **86** | • recognize and address urgent health and safety needs of caregivers; |  |  |  |
|  | **87** | • consider that caregivers may feel significant stress, and that past and current trauma may be affecting the caregiver’s emotional state and behaviour; |  |  |  |
|  | **88** | • establish a rapport with caregivers and encourage their active engagement and participation in the provision of care, whenever safe and appropriate; |  |  |  |
|  | **89** | • treat caregivers with respect, without being confrontational; |  |  |  |
|  | **90** | • avoid blaming or stigmatizing caregivers and identify what they are doing well; |  |  |  |
|  | **91** | • take care not to allow relationships with other family members to interfere with the ability to consider maltreatment; |  |  |  |
|  | **92** | • ensure that the caregivers understand the potential health consequences of the abuse or neglect, the significance and possible side-effects of any interventions, and options and rights in consenting to or refusing treatment for the child or adolescent as well as the limitations of confidentiality and obligations to report. |  |  |  |
|  | **93** | • carefully consider what information can be shared with whom in order to avoid placing the child or non-offending caregiver at risk of further harm. |  |  |  |
|  |  |  |  |  |  |
|  | **E. Guiding Principles** | |  |  |  |
|  |  | **Principle of best interest of child/adolescent** |  |  |  |
|  | **94** | **Protect and promote safety**: promoting and protecting the physical and emotional safety of the child or adolescent must be the primary consideration throughout the course of care. This means that, with the participation from the child and adolescent and their non-offending caregivers, as appropriate depending on the age and the wishes of the child, health-care providers need to **consider all potential harms** and **take or choose actions that will minimize the negative consequences** on the child or adolescent, including the likelihood of the maltreatment continuing. |  |  |  |
|  | **95** | **Provide sensitive care:** children and adolescents who disclose maltreatment including abuse need to be listened to attentively, without interpreting or judging their account, even when it might differ from that of the accompanying caregivers. Children and adolescents should be offered an empathetic and non-judgemental response that reassures them that they are not to blame for the maltreatment including abuse and that they have acted appropriately in disclosing it. |  |  |  |
|  | **96** | **Protect and promote privacy and confidentiality:** protecting privacy during care and confidential handling of all collected information is of particular importance to promoting the safety of the child or adolescent. This means that during consultation and examination, only those who need to be present in the room (also to prioritize the safety and the wellbeing of the child or adolescent) should be allowed. For safety reasons, children and adolescents should be interviewed on their own, separately from the caregiver. Information collected from interviews and examination should be shared on a need-to-know basis and only after obtaining informed consent and assent from the child or adolescent and/or caregivers, as appropriate. Where there are limits to confidentiality, including any obligations to report incidents, these should be explained to children/adolescents and their caregivers at the beginning of care provision. Collected information should be stored securely (e.g. protected by key or password). |  |  |  |
|  |  | **Principle of evolving capacity of child/adolescent** |  |  |  |
|  | **97** | **Provide information that is appropriate to age and developmental stage as well as to other considerations** (e.g. sex, race, ethnicity, religion, sexual orientation, gender identity, disability and socioeconomic status). This requires tailoring the information that is offered and how it is delivered (e.g. in choice of words or language, use of visual aids) to the child’s or adolescent’s age and developmental stage, including their cognitive, behavioural and emotional maturity to understand the information. |  |  |  |
|  | **98** | **Seek informed consent and assent as appropriate to the child’s or adolescent’s age and evolving capacity and the legal age of consent** for obtaining clinical care for all decisions and actions to be taken. Where the child or adolescent is below the legal age of consent, it may still be in their best interests to seek informed consent. For example, in some situations, adolescents may be deterred from seeking care where consent is required from their parents or legal guardians. Recognizing this, in some settings, older adolescents are able to provide informed consent in lieu of, or in addition to, their parents or legal guardians. Moreover, the Committee for the Rights of the Child (CRC) recognizes that, in accordance with evolving capacities, children have the right to access confidential counselling or advice and information without the consent of their parents or legal guardians.12 In situations where it is assessed to be in the best interests of the adolescents who are in need of care, and based on their preferences, health-care providers may consider whether to involve the parents or legal guardians. |  |  |  |
|  | **99** | **Respect the autonomy and wishes of children or adolescents** (e.g. not forcing them to give information or be examined) while balancing this with the need to protect their best interests (e.g. protect and promote their safety). In situations where a child’s or adolescent ́s wishes cannot be prioritized; the reasons should be explained to the child or adolescent before further steps are taken. |  |  |  |
|  | **100** | **Offer choices** in the course of the medical care. |  |  |  |
|  |  | **Principle of non-discrimination** |  |  |  |
|  | **101** | This principle requires that **all** children and adolescents should be offered quality care, **irrespective of their sex, race, ethnicity, religion, sexual orientation, gender identity, disability or socioeconomic status**. Health-care providers need to recognize and take into account gender and other social inequalities that can disproportionately increase vulnerabilities to maltreatment and pose barriers in access to services for some groups over others. Therefore, attention should be paid to the specific needs of groups in special or vulnerable situations – for example, adolescent girls from poor communities, children or adolescents with disabilities, adolescents who are part of the lesbian, gay, bisexual, transgender or intersex (LGBTI) communities, or adolescents that are part of ethnic minorities and indigenous groups. |  |  |  |
|  |  | **Principle of participation** |  |  |  |
|  | **102** | Children and adolescents have **a right to participate in decisions that have implications for their lives, in accordance with their evolving capacities. I**n practice this means they should be asked what they think and have their opinions respected and taken into account when decisions are being made in relation to clinical care being offered to them. Moreover, young people generally want to be consulted and engaged and to meaningfully participate in the design and delivery of health services that affect them. Interventions with children exposed to abuse and neglect includes **assisting the improvement of the child-caregiver relationship (when safe and appropriate!)** to restore a sense of safety and trust and providing caregivers with positive parenting, coping, and life-skills essential for healthy family functioning. Thus, **engaging caregivers’ participation in intervention** is critical to the child’s healing from the trauma of maltreatment and promoting the child’s healthy development and resilience. **Being respectful towards caregivers and communicating with empathy,** recognizing how their difficult current and past adversities and circumstances may affect their parenting, is essential to building trust and supporting their engagement in the provision of care for their children |  |  |  |
|  | **Total # items scored = 101** |  |  |  |  |

# Supplement 8: Identified definitions of child sexual abuse from WHO 2017 and included national clinical practice guidelines (n=24)

| S.No. | CGPs | Country | Definition |
| --- | --- | --- | --- |
| 1. | WHO 2017 definition | International | The involvement of a child or an adolescent in sexual activity that he or she does not fully comprehend and is unable to give informed consent to, or for which the child or adolescent is not developmentally prepared and cannot give consent, or that violates the laws or social taboos of society. Children can be sexually abused by both adults and other children who are – by virtue of their age or stage of development – in a position of responsibility or trust or power over the victim. It includes incest which involves abuse by a family member or close relative. Sexual abuse involves the intent to gratify or satisfy the needs of the perpetrator or another third party including that of seeking power over the child. Adolescents may also experience sexual abuse at the hands of their peers, including in the context of dating or intimate relationships.  Three types of child sexual abuse are often distinguished: (i) non-contact sexual abuse (e.g. threats of sexual abuse, verbal sexual harassment, sexual solicitation, indecent exposure, exposing the child to pornography); (ii) contact sexual abuse involving sexual intercourse (i.e. sexual assault or rape – see below); and (iii) contact sexual abuse excluding sexual intercourse but involving other acts such as inappropriate touching, fondling, and kissing. Child sexual abuse is often carried out without physical force, but rather with manipulation (e.g. psychological, emotional, or material). It may occur on a frequent basis over weeks or even years, as repeated episodes that become more invasive over time, and it can also occur on a single occasion. |
| 2. | ([A. Ciresa-König et al., 2021](#_ENREF_1)) | Austria | No definition is identified within this document. |
| 3. | ([Keygnaert I. et al., 2021](#_ENREF_14)) | Belgium | No definition is identified within this document. |
| 4. | ([N. Dekker, K. SmetS, & PeremaNS, 2014](#_ENREF_21)) | Belgium | “Sexual abuse of a child is defined as the child's involvement in sexual activities, which he or she does not fully comprehend and is inappropriate to consent to, or for which the child is not developed enough, or which violates the laws or social taboos of the child. community violates. Children can be sexually abused by both adults and other children who, because of their age or stage of development, are in a position of responsibility, trust or power towards the victim.” |
| 5. | ([Croatian_Government, 2014](#_ENREF_6)) | Croatia | “Sexual abuse is participation in sexual activities with a child who is younger than the legal age for sexual activities and participation in sexual activities with a child in which coercion, force or threats are used or a position of trust, authority or influence over the child is abused (in the family or outside family) or the child's particularly vulnerable situation is abused. In addition to sexual abuse, sexual violence also includes other forms of sexual exploitation, a number of acts related to child prostitution and child pornography, participation of children in pornographic performances, luring and recruiting children for sexual purposes, and aiding or abetting any of these acts.” |
| 6. | ([Lassen, Christiansen, Jørgensen, & Berg, 2022](#_ENREF_16)) | Denmark | “Definition of sexual abuse: It is sexual abuse when a child is involved in sexual activities for which he cannot understand the scope, is not developmentally ready for, and therefore cannot give permission for, and/or activities of this nature that exceed society's social or legal norms (Kempe 1971).”  In Denmark, the sexual minimum age is 15.  Sexual assault includes the following types:  1. Assault without physical contact between the child/youth and the offender. There has been solicitation for something sexual, been sexually photographed fully or partially naked, viewed pornographic images or pornographic films with adults, or watched a person expose themselves or masturbate. Attention to grooming and virtual violation.  2. Assault with physical contact between the child/youth and the offender. The child has been kissed or touched in a sexual way with or without clothes. Have had to touch an offender's genitals or had their clothes removed.  3. Assault where intercourse has been attempted or intercourse completed/completed, incl. digital/instrumental penetration. Possible penetration with risk of mucosal contact. Oral, vaginal or anal. |
| 7. | ([Luoma, Joki-Erkkilä, & Taskinen, 2021](#_ENREF_19)) | Finland | No definition is identified within this document. |
| 8. | ([Dhénain, 2014](#_ENREF_7); [HAS, 2017](#_ENREF_10)) | France | Sexual violence classified into 3 categories: i) according to the Third National Incidence Study definition: sexual violence with rape; with touching; other or unknown; ii) stricter than the Third National Incidence Study definition: most often limited to rape; broader than the Third National Incidence Study definition: when non-contact violence was included (e.g. exhibitionism).  Sexual abuse of a minor is defined as forcing or inducing the minor to engage in sexual activity. This constitutes an attack on the physical and psychological integrity of the minor, as the minor does not have sufficient maturity and development to understand the meaning and/or consequences. A minor is defined as a person under the age of 18.  Intra-family sexual abuse or incest against a minor is defined as sexual abuse committed within the family on the person of a minor by an ascendant, a brother, a sister or any other person, including a cohabitant of a family member, having de jure or de facto authority over the victim (according to the law of 08/02/2010). |
| 9. | ([Blesken et al., 2022](#_ENREF_5)) | Germany | No definition is identified within this document. |
| 10. | ([Keller et al., 2016](#_ENREF_13)) | Hungary | "Child abuse and neglect (maltreatment) includes all forms of physical and/or emotional maltreatment, sexual abuse, neglect or negligent treatment, commercial or other exploitation that have a real impact on the child's health, survival, development or dignity or results in potential harm in a relationship based on responsibility, trust, or power.” |
| 11. | ([Team, 2018](#_ENREF_25)) | Ireland | Child victims aged 16 years or younger cannot consent in law to a ‘sexual act’ which is defined as: sexual intercourse between persons not married to each other, or buggery between persons not married to each other, or aggravated sexual assault, or ‘rape under section 4’ meaning a sexual assault that includes: penetration (however slight) of the anus or mouth by the penis, or penetration (however slight) of the vagina by any object held or manipulated by another person. It may be a defence for a person who engages in any of the above sexual acts with a child victim aged 15 or 16 years, that the child victim consented, provided that the person is younger or less than two years older than the child victim, and was not a person in authority in respect of the child victim, or in an intimidatory or exploitative relationship with the child victim.6 Child victims aged 14 years or younger cannot consent in law to any of the aforementioned sexual acts and, in addition, cannot consent to acts amounting to sexual assault or indecent assault.  The World Health Organisation defines child sexual abuse as the “involvement of a child in sexual activity that he or she does not fully comprehend, is unable to give informed consent to, or for which the child is not developmentally prepared, or else that violates the laws or social taboos of society” and further notes that “children can be sexually abused by both adults and other children who are – by virtue of their age or stage of development – in a position of responsibility, trust or power over the victim” . |
| 12. | ([health.gov.il, 2022](#_ENREF_11)) | Israel | Act of sodomy - inserting an organ of the body, or an object, into a person's anus, or inserting a genital into the mouth of a person Those that reach a student, a patient or a person within a working relationship are considered sexual harassment even if it should be noted that even in colloquial terms inserting a genital organ into a person's mouth is usually called "oral sex", after all that from a legal point of view it is an act of sodomy. An act of sodomy committed on a person under the circumstances specified in the definition of rape is considered as rape: an act of sodomy on a minor between the ages of 14 and 16; an act of sodomy on a minor between the ages of 16 and because these people depend on the people they come in contact with. A student depends on a teacher, a patient on a doctor or a psychologist. While exploiting relationships of dependence, malice, education or supervision, a sodomy act on an adult while exploiting malice in work relationships or in service.  Indecent act is any act for sexual stimulation, satisfaction or humiliation. It will be considered a crime when it is done without the person's consent; done under the circumstances listed in the definition of rape; done under the definition of prohibited ground by consent; Other; It is done on a minor who has turned 14, taking advantage of relationships of dependence, control, education, supervision, work or service; done in public; To a minor under the age of 16. |
| 13. | ([Angeletti et al., 2020](#_ENREF_4)) | Italy | We speak of sexual abuse when a child is involved in sexual activities that he cannot understand, for which he is psychologically unprepared and for which he cannot give his consent and / or who violate laws or social taboos. Sexual activities can include all forms of orogenital, genital or anal contact with the child, or abuse without direct contact such as exhibitionism, voyeurism, or the child's involvement in the production of pornography. Sexual abuse includes a spectrum of activity ranging from rape to less intrusive sexual abuse. (American Aca demy of Pediatrics, 1999). |
| 14. | ([Longo, Cremonesi, Pitidis, Santi, & Sparaco, 2017](#_ENREF_18)) | Italy | “Sexual abuse: this refers to the involvement of a minor in sexual activities (including pornography) even if not characterized by explicit violence. More precisely, it lies: “any act in which an adult uses his power over a child to obtain sexual gratification by taking advantage of the child's vulnerability and / or trust”. Peculiarities that characterize child sexual abuse include the following considerations:  • violence or physical force is rarely used;  the perpetrator is usually a known person;  • sexual abuse in the child takes place over a long period of time;  • frequently the episodes are repeated and more and more intrusive; the victim is involved in acts and situations unsuitable for his age and experience”. |
| 15. | ([Orsini, Pagano, Raciti, & Seniga, 2014](#_ENREF_23)) | Italy | “Sexual abuse of children and adolescents Involvement of a minor by a dominant adult partner in sexual activities, even if  not characterised by explicit violence (Cismai, 2001). Child sexual abuse encompasses a broad spectrum of sexual offences in which children under the age of 18 are regarded as victims (see sexual majority). The Council of Europe Convention on the Protection of Children against Sexual Exploitation and Sexual Abuse (2007) offers a definition of child sexual abuse in Article 18:  a) engaging in sexual activities with a person under the age of 18 who, according to local law, has not reached the legal age to engage in such activities; b) engaging in sexual activities with a minor where: -- such activities take place by means of coercive methods, the use of force or threats;  -- the violence takes place 'by abusing a recognised position of trust, authority or influence over the child, including in cases where this occurs within the family';  -- the abuse occurs if the child is in a particularly vulnerable situation, due to mental or physical disabilities or in a situation of  dependence.  Online sexual abuse  Involvement of a minor in sexual activities by means of the tools offered by new technologies.  Online sexual abuse includes:  -- activities performed in the context of offline sexual abuse photographed or filmed and disseminated online;  -- producing, distributing, downloading and displaying material concerning sexual violence against minors (images and videos);  -- send online requests to children and young people to make the material themselves;  -- engaging in sexual chats or other online sexual activities or arranging a meeting in real life to pursue a sexual purpose, (see  Online Grooming);  -- encourage or facilitate any of the activities described” |
| 16. | ([Dulkys, 2021](#_ENREF_8)) | Lithuania | 3.3 . Sexual violence - intentional criminal acts, as defined in Chapter XXI of the Criminal Code of the Republic of Lithuania "Crimes and misdemeanors against the freedom and inviolability of human sexual self-determination". (Chapter I, page. 3) |
| 17. | Adomaitieny, 2019 | Lithuania | “6. Sexual rape - forcible satisfaction of sexual passion between the same person or persons of a different gender other than Article 149 of the Criminal Code of the Republic of Lithuania. in the prescribed ways (without vaginal intercourse). Sexual passion is satisfied against the will of the victim through anal, by oral or other physical contact (sexual passion is satisfied by the stimulation of a man or female genital organs, touching the body of another person with the genital organ, inserting into the victim anus, genitals, fingers or objects, etc.).  7. Forced to have sexual intercourse is coercive (threatened to use violence, using other mental coercion or taking advantage of a person's addiction to of the perpetrator, except for the use of physical violence) satisfaction of sexual passion between that in any form of sexual intercourse between persons of the same or opposite sex: both in a natural way, both anal and oral ways, as well as in such forms that does not include intervention on the victim's body, such as masturbation by touching the penis body of another person's buttocks.” |
| 18. | ([Adlienÿ et al., 2018](#_ENREF_2)) | Lithuania | “3. Sexual violence - intentional criminal acts, as defined by the Criminal Code of the Republic of Lithuania In Chapter XXI "Crimes and misdemeanors against the freedom of sexual self-determination of a person and does not affect our dignity", committed against a child, as well as profiting from a child prostitution, engaging a child in prostitution or engaging in participation in a pornographic event, showing pornography to a child, forcing a child to engage in prostitution, exploiting pornography or possessing pornographic content that depicts a child or presents a person as a child, or involving a child in sexual slavery and other forms of child sexual exploitation.” |
| 19. | ([Moldova, 2021](#_ENREF_20)) | Moldova | “Sexual Violence (WHO) - any sexual act, attempted sexual act, unwanted sexual comments or advances, trafficking actions or any other kind of actions directed against a person's sexuality through coercion by another person, regardless of their relationship with the victim and in any setting, including, but not limited to, at home and at work. It includes:  Rape/attempted rape (art. 171 Penal Code): represents a sexual relationship committed by physical or mental coercion of the person or taking advantage of his inability to defend himself or to express his will. Sexual intercourse involves vaginal penetration. Efforts to rape someone that do not result in penetration are considered attempted rape.  Violent actions of a sexual nature (art. 172 Penal Code): homosexuality or the satisfaction of sexual desire in perverse forms, committed by physical or mental coercion of the person, or taking advantage of his inability to defend himself or express his will. Homosexual activities assume that the aggressor and the victim are of the same sex, and perversions include oral or anal penetration.  Sexual harassment (art. 173 Criminal Code): physical, verbal or non-verbal behaviour, which damages the dignity of the person, or creates an unpleasant, hostile, degrading, humiliating, discriminatory or insulting atmosphere, with the aim of causing a person to have sexual relations or other unwanted sexual actions, committed by threat, coercion, blackmail.  Sexual intercourse with a person who has not reached the age of 16 (art. 174 of the Criminal Code): sexual intercourse other than rape, acts of vaginal, anal or oral penetration and others, committed on a person who was known with certainty to have not reached the age of 16”. |
| 20. | ([FMG, 2021](#_ENREF_9)) | Netherlands | No definition is identified within this document. |
| 21. | ([Teeuw, Langendam, & Boseloos, 2016](#_ENREF_26)) | Netherlands | What is the definition of sexual abuse in children?  Sexual abuse of children occurs in the following cases:  - Child under 12: any sexual contact, both sexual penetration and groping, whether or not accompanied by coercion or force (excluding sexual play between children under 12 of the same age (less than 3 years difference) and developmental level).  - Child between 12-16 years: any sexual contact, both penetration and groping in principle whether or not accompanied by coercion or force, unless it is a voluntary relationship between equal sexual partners of approximately equal age.  - Child between 16-18 years: any sexual contact, both penetration and groping, involving: o coercion/violence o gifts of money or goods   - if the perpetrator is in a special capacity (parents, carers, teachers, social workers, etc.) - if the minor prostitutes himself/herself or works in game shows - if the minor is forced by a third party to have sex with someone   - Child under 16: if pornographic images are shown or if the child has been present during sex between others, or if an appointment is made with the child for the purpose of having sex.  - Child under 18: if child pornographic images are made of them or any other form of sexual exploitation such as child prostitution takes place.  NB/ Coercion also refers to psychological coercion resulting from a relationship of dependency of the minor on an adult. |
| 22. | ([helsebiblioteket, 2021](#_ENREF_12)) | Norway | No definition is identified within this document. |
| 23. | ([Lips, Wopmann, Jud, & Falta, 2020](#_ENREF_17)) | Switzerland | The term sexual abuse refers to the involvement of children or young people in sexual acts to which they cannot give their consent or which they do not understand due to their level of development, or in sexual acts that violate social taboos.  Sexual abuse includes many forms such as  (not exhaustive):  - Assaults in electronic media  - exhibitionism, voyeurism  - Touching the intimate area  - the desire to be masturbated or caressed  - sexual assault  - exposure to pornography  - Introduction to Prostitution  - anal, oral and vaginal penetration |
| 24. | ([NICE, 2019](#_ENREF_22)) | United Kingdom | Sexual abuse: Involves forcing or enticing a child or young person to take part in sexual activities, not necessarily involving a high level of violence, whether or not the child is aware of what is happening. The activities may involve physical contact, including assault by penetration (for example, rape or oral sex) or non-penetrative acts such as masturbation, kissing, rubbing and touching outside of clothing. They may also include non-contact activities, such as involving children in looking at, or in the production of, sexual images, watching sexual activities, encouraging children to behave in sexually inappropriate ways, or grooming a child in preparation for abuse (including through the internet). Sexual abuse is not solely perpetrated by adult males. Women can also commit acts of sexual abuse, as can other children.  Child sexual exploitation is a form of child sexual abuse. It occurs where an individual or group takes advantage of an imbalance of power to coerce, manipulate or deceive a child or young person under the age of 18 into sexual activity (a) in exchange for something the victim needs or wants, and/or (b) for the financial advantage or increased status of the perpetrator or facilitator. The victim may have been sexually exploited even if the sexual activity appears consensual. Child sexual exploitation does not always involve physical contact; it can also occur through the use of technology. |
| 25. | ([RCPCH, 2015](#_ENREF_24)) | United Kingdom | RCPCH “Purple Book 2015” begin with citing the Working Together to Safeguard Children (2013) CSA definition:  ‘Sexual abuse involves forcing or enticing a child or young person to take part in sexual activities, not necessarily involving a high level of violence, whether or not the child is aware of what is happening. The activities may involve physical contact, including assault by penetration (for example, rape or oral sex) or non-penetrative acts such as masturbation, kissing, rubbing and touching outside of clothing. They may also include non-contact activities, such as involving children in looking at, or in the production of, sexual images, watching sexual activities, encouraging children to behave in sexually inappropriate ways, or grooming a child in preparation for abuse (including via the internet). Sexual abuse is not solely perpetrated by adult males. Women can also commit acts of sexual abuse, as can other children.’  Many types of sexual abuse do not result in injuries and many children who allege vaginal or anal abuse have no clinical signs on examination. In this publication, the term ‘child sexual abuse’ or ‘CSA’ refers to physical contact that involves penetrative or non-penetrative activities that might cause anogenital injuries or result in either the diagnosis of a sexually transmitted infection (STI) or pregnancy in a child under 18 years of age. Child sexual exploitation (CSE) and child trafficking are abusive and may involve sexual abuse. Female genital mutilation (FGM), though abusive in terms of human rights, is a cultural phenomenon rather than a result of sexual abuse. |

Supplement 9 - Figure 4: Distribution of standardized scores by domain for the 24 NCPGs
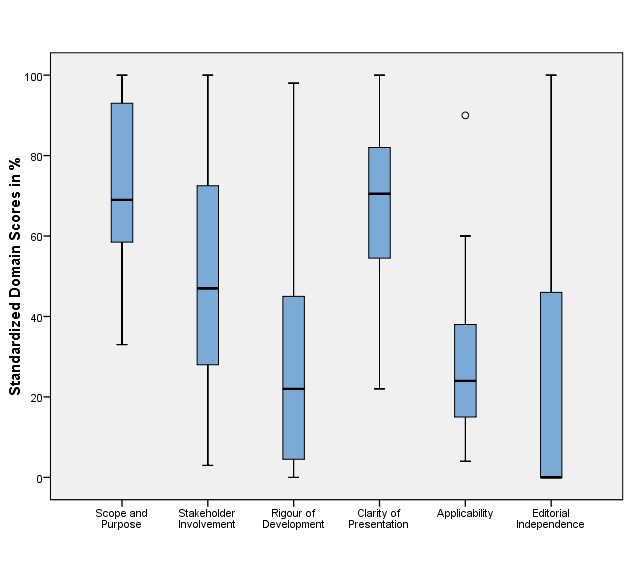


# Supplement 10 - Table 4: Child sexual abuse guidelines quality changes over the years from 2014-2017 and 2018-2022

| Domain | Guidelines from 2014-2017  Mean (SD) | Guidelines from 2018-2022  Mean (SD) | *P-Value |
| --- | --- | --- | --- |
| Scope and Purpose | 82·3 (16·7) | 66·8 (23·1) | 0·106 |
| Stakeholder Involvement | 68·5 (17·4) | 43 (27·5) | 0·027* |
| Rigour of Development | 40·3 (31·1) | 24·6 (28·7) | 0·233 |
| Clarity of Presentation | 63·1 (25) | 70·6 (16·3) | 0·382 |
| Applicability | 25 (12·9) | 30·1 (23) | 0·562 |
| Editorial Independence | 26·1 (25·4) | 21·6 (35·1) | 0·752 |
